# Tibetan medicine Baxiaga: a systematic review of its historical herbalogical investigation, phytochemical constituents, and pharmacological activities

**DOI:** 10.3389/fphar.2026.1787813

**Published:** 2026-03-27

**Authors:** Nengqiao Fang, Hanyu Lu, Huimin Gao, Jing Ma, Renzhen Wangjia, Hang Chen, Jinsong Su, Yi Zhang

**Affiliations:** 1 Chinese Medicine Germplasm Resources Innovation and Effective Uses Key Laboratory of Sichuan Province, School of Pharmacy, Chengdu University of Traditional Chinese Medicine, Chengdu, Sichuan, China; 2 Ethnic Medicine Academic Heritage Innovation Research Center/Meishan Hospital of Chengdu University of Traditional Chinese Medicine, Chengdu University of Traditional Chinese Medicine, Chengdu, Sichuan, China; 3 State Key Laboratory of Southwestern Chinese Medicine Resources, Innovative Institute of Chinese Medicine and Pharmacy, Chengdu University of Traditional Chinese Medicine, Chengdu, Sichuan, China; 4 College of Pharmacy, Qinghai Minzu University, Xining, Qinghai, China; 5 Ganzi Agricultural Sciences Institute, Ganzi, Sichuan, China

**Keywords:** Baxiaga, pharmacological activities, phytochemistry, Tibetan medicine, traditional medicinal uses

## Abstract

**Background:**

Baxiaga (བ་ཤ་ཀ) is a typical Tibetan medicinal species of multiple origin. These plants have been used in traditional Tibetan medicine for centuries and are currently used to treat various types of fever, such as Chiba fever, liver fever, reduce inflammation, relieve pain, and heal other diseases.

**Objective:**

This review aims to provide a comprehensive analysis of the traditional uses of the baxiaga series of plants (*Justicia adhatoda* L., *Veronica ciliata* Fisch., *Veronica eriogyne* H.J.P. WinkI., *Corydalis impatiens* (Pall.) Fisch. ex DC., and *Corydalis crispa* Prain) as well as their phytochemical and pharmacological studies; it also provides a scientific basis for the clinical application of Baxiaga and promotes the development of its pharmaceutical preparations.

**Materials and Methods:**

This review performed an extensive database search to collect detailed information about baxiaga and the literature was systematically interpreted, analyzed, and documented.

**Results:**

This review summarizes the traditional usage of five plants in Tibetan medicine baxiaga, which includes 195 chemical constituents such as alkaloids, flavonoids, terpenoids, and iridoid glycosides. This research elaborates on the pharmacological activities and mechanisms of these medicinal plants, along with discussions on multi-origin and quality control issues, while also proposing future research directions.

**Conclusion:**

This study presents a comprehensive analysis of the five medicinal plants commonly used in baxiaga. Our analysis highlights the significant value of baxiaga as an ethnomedicinal resource and reveals that its pharmacological activity is mainly attributed to its alkaloid. These alkaloids demonstrate anti-inflammatory, antioxidant and hepatoprotective, activities. However, current studies on the medicinal effects, active ingredient content, quality control, and clinical applications of baxiaga have limitations.

## Graphical Abstract

**Figure:**
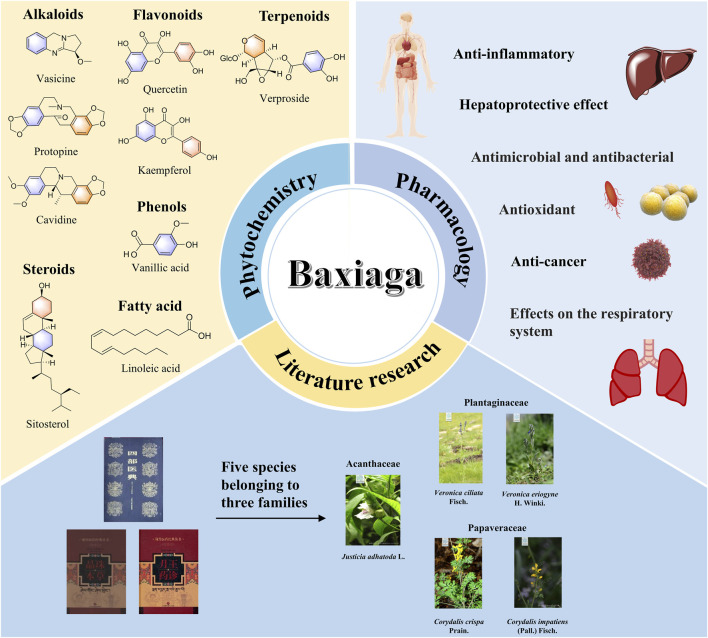

## 1 Introduction

Baxiaga is one of the commonly employed medicinal materials in Tibetan medicine. Its morphological characteristics and plant origin were first recorded in the 17th century in *Tara Materia Medic* (Mao, 2016a), which states “As it is called, the medicine of baxiaga is woody, grows in the rivers and forests of the south, with tall trunks and thick leaves, and the flowers are like a bird’s beak. In *Blue Glaze* (Mao, 2012a), baxiaga is classified into two categories: the upper category, belonging to trees with various flower colors, such as white, red, blue, and yellow; and the lower category, belonging to grasses that can grow everywhere. In the absence of upper baxiaga, the lower category can be used as a substitute. According to the *Crystal Beads*, Baxiaga possesses therapeutic effects for fever diseases, biliary disorders, liver diseases, blood disorders, and wound healing. Clinically, it is widely used in the treatment of rheumatoid arthritis, hepatobiliary diseases, gastric ulcers, hypertension, and other conditions (Wangjia et al., 2021).

Baxiaga plants are primarily found on the Tibetan Plateau in Southwestern China and are widely used in Tibetan medicine. Before the extraction of baxiaga compounds, the whole botanical drugs or preparations were used in traditional formulations. Some traditional compound formulations are still in use today. What are the pharmacologically active constituents in the source plants? What is the modern scientific basis for these traditional effects? Although research on Baxiaga has gradually addressed these questions, existing findings remain fragmented and predominantly focused on individual plant species, lacking systematic review. These issues continue to hinder the development of modern formulations of Baxiaga. Furthermore, advancing the scientific understanding and translational application of Baxiaga encounters a fundamental yet often overlooked challenge inherent to its identity in Tibetan medicine: its multi-source nature. Tibetan medicine “Baxiaga” is not a single species but an important medicinal complex. According to classical texts such as The *Crystal Beads* and *Four Medical Treatises*, it is traditionally classified into three categories: woody (superior grade), herbaceous (medium/inferior grade), and substitutes. This complex encompasses plants from multiple genera, including *Justicia* (Acanthaceae), *Veronica* (Plantaginaceae), and *Corydalis* (Papaveraceae). Different botanical sources are likely to yield distinct alkaloid profiles, which can lead to inconsistent research outcomes and difficulties in replicating therapeutic efficacy. Pharmacological activities observed in studies using one specific source may not be directly attributable to a universally defined “Baxiaga.” Furthermore, both historical and contemporary clinical experiences attributed to “Baxiaga” may originate from different plant species, making it exceptionally challenging to correlate traditional uses with specific phytochemical compositions or modern mechanistic data.

This review not only provides a detailed summary of the studies on the herbal and resource distribution, traditional uses, phytochemistry, pharmacological effects, and toxicity of baxiaga. But also rigorously evaluate the existing evidence through the lens of its phytochemical and pharmacological diversity. And we highlights the current limitations in research on Baxiaga and proposes potential solutions based on our analysis, thereby offering valuable insights to support future studies on Baxiaga and the development of related new drugs. Additionally, it aims to promote the safe and rational use of Tibetan medicines in clinical practice.

## 2 Experimental methods

This review employed search strings such as (“Adhatoda vasica” OR “Justicia adhatoda” OR “Baxiaga” OR “Popona”) AND (“alkaloids” OR “vasicine” OR “phytochemistry”), as well as (“Justicia adhatoda” OR “Veronica ciliata”) AND (“anti-inflammatory” OR “hepatoprotective” OR “pharmacology”). Literature retrieval was conducted by searching classical texts and databases including Google Scholar, Web of Science, SciFinder, Baidu Scholar, PubMed, and CNKI.

## 3 Traditional uses and ethnopharmacological rationale

### 3.1 Traditional classification

**FIGURE 1:**
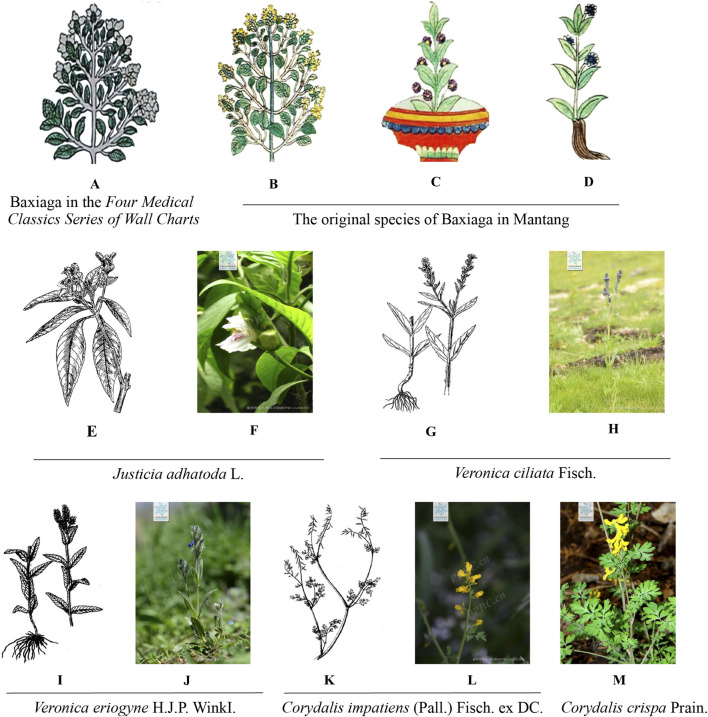
Results of variety research and sorting of Baxiaga. **(A)** Baxiaga in the *Four Medical Classics Series of Wall Charts*; **(B–D)** The original species of Baxiaga in Mantang; **(E,F)** *Justicia adhatoda* L; **(G,H)** *Veronica ciliata* Fisch.; **(I,J)** *Veronica eriogyne* H.J.P. WinkI.; **(K,L)** *Corydalis impatiens* (Pall.) Fisch. ex DC.; **(M)** *Corydalis crispa* Prain. The above pictures **(F,H,J,L,M)** were accessed through https://www.iplant.cn/.

As shown in Table 1, Baxiaga originates from five primary botanical sources, and is also known by aliases such as “Yazuihua” and “Popona” in herbal medicine contexts. The *Crystal Beads* (DiMa and Mao, 1986) and *Four Medical Treatises* (YuTuo, 1982) further classify baxiaga species into three categories: woody, herbaceous, and substitutes. Traditional woody baxiaga, considered the top category, has a bitter taste and cool properties. It reduces swelling and pain, clears heat and toxicity, treats blood and liver fever, and cures gallbladder and Chiba disease. However, it is not grown in Tibetan areas. Herbaceous, or inferior, baxiaga, which is also known as dongnaduanchi, emengdongchi, or baxiagamanba, is planted in Tibet. It has small roots, long stems, and thick cyan leaves covered with villi. The flowers are yellow, blue, white, or purple, and this type has superior and inferior varieties. Dongnaduanchi has a bitter and slightly sweet taste and can be used to clear heat, stop bleeding, heal sores, regenerate muscles, and reduce fever from sores. When these plants are unavailable, they can be replaced by yudongsaiguo, which has small leaves, a slender and pliable stem in quadrangular shape, and yellow flowers. It also has a bitter taste and is effective in reducing swelling. The classic works of Tibetan medicine, namely, the *Crystal Beads* (DiMa and Mao, 1986), the *Four Medical Classics* (YuTuo, 1982), and various later Tibetan medical publications, have summarized six species of plants used as Tibetan medicine baxiaga according to previous authors’ classification methods. The original plant species of baxiaga is *Justicia adhatoda* L., which belongs to the genus *Justicia* and the family Acanthaceae. The main original plants of dongnaduanchi are identified as *Veronica ciliata* Fisch. and *Veronica eriogyne* H.J.P WinkI. of the genus *Veronica* in the family Plantaginaceae. The original plants of yudongsaiguo belong to the genus *Corydalis*, specifically *Corydalis impatiens* (Pall.) Fisch. ex DC. and *Corydalis crispa* Prain (Figure 1).

**TABLE 1:** The historical herbalogical investigation of Baxiaga.

Botanical name	Family name	Categories	Medicinal material name	References
*Justicia adhatoda* L.	Acanthaceae	Baxiaga	Yazuihua Paxiaga Waxiaga Baxiaga Yazuihauye	(Luo, 2004; Northwest Institute of Plateau Biology, Chinese Academy of Sciences, 1991) Editorial committee (2002) SiChuan Medical Products Administration (2020) Yunnan Medical Products Administration (2006)
*Veronica ciliata* Fisch.	Plantaginaceae	Dongnaduanchi	Popona Dongnaduanchi Waxiaga	Luo (2004) Northwest Institute of Plateau Biology, Chinese Academy of Sciences (1991) QingHai Medical Products Administration (2019)
*Veronica eriogyne* H.J.P. WinkI.	Plantaginaceae	Dongnaduanchi	Popona Dongnaduanchi Dangnadongchi	(Luo, 2004; Editorial committee, 2002) Northwest Institute of Plateau Biology, Chinese Academy of Sciences (1991) SiChuan Medical Products Administration (2014)
*Corydalis impatiens* (Pall.) Fisch. ex DC.	Papaveraceae	Yudongsaiguo	Paxiaga Demuwaxiaga Saibeizijin	Northwest Institute of Plateau Biology, Chinese Academy of Sciences (1991) QingHai Medical Products Administration (2019) (SiChuan Medical Products Administration, 2011)
*Corydalis crispa* Prain.	Papaveraceae	Yudongsaiguo	Zhoubohuangjin	The Tibet Autonomous Region Food and Drug Administration (2012)

### 3.2 Parts used, usage and treatment of diseases

As detailed in Table 2, this study summarizes the properties, medicinal parts, and traditional indications of the five different botanical sources of Baxiaga. The single herb Baxiga is primarily used for the treatment of inflammatory diseases. Most baxiaga plants use the whole plant as medicinal parts, whereas *J. adhatoda* uses stems, leaves, or branches. For instance, the levels of vasicine **1** and vasicinone **2** are highest in *J. adhatoda* leaves, lower in stems, and lowest in roots. The stems, leaves, or branches of *J. adhatoda*, which are cold and cool in properties, are effective in clearing heat, removing toxicity, reducing inflammation, and relieving pain. They are widely used to treat blood-heat diseases, liver and gallbladder fever, and Chiba disease and cure bruises and sores. *V. ciliata* is used as medicine in its entirety for treating hepatitis, cholecystitis, blood fever, trauma, and swelling. *V. eriogyne*, which tastes bitter and sweet, is known to stop bleeding and cure sores. The whole plant of *C. crispa* can clear heat, cool the blood, and relieve pain. Although plants in the baxiaga group are often made into pills, powders, or decoctions for internal use, some can also be used externally. The whole plant of *C. impatiens* has a medicinal value; it clears heat and treats various kinds of feverish diseases when taken orally, while it reduces swelling, dissipates blood stasis, and treats sores and abscesses when applied externally (Table 2) (Northwest Institute of Plateau Biology, Chinese Academy of Sciences, 2019; Luo, 2018; Editorial Committee, 2002).

**TABLE 2:** The traditional uses of Baxiaga.

Botanical name	Part	Taste and properties	Usage	Traditional uses	Distributions	References
*Justicia adhatoda* L.	The stems, leaves, or branches	Bitter, cold and cool	Internal use: 15∼20 g	Clear heat and removing toxins Reducing inflammation and relieving pain Blood-heat diseases Liver and gallbladder fever Chiba disease in Tibetan medicine Cure bruises and sores Treatment of falls	Growth altitude: below 1,500 m China (Guangdong, Guangxi, Hainan, Yunnan) Indian Nepal Pakistan Myanmar Bangladesh Central America Mexico	(Luo, 2018; Mao, 2012a, 2016; YuTuo, 1982)
*Veronica ciliata* Fisch.	The whole plant	Bitter, cold and cool	Internal use: 2∼9 g	Clear heat and removing toxins Reducing inflammation and relieving pain Hemostatic and myogenic muscle Neurasthenia Cholecystitis Liver and gallbladder fever Cure bruises and sores Blood-heat diseases	Growth altitude: 2,500∼4,500 m China (Qinghai, Tibet, Yunnan, Sichuan, Gansu, Shaanxi, Neimenggu) Russia The Himalayas Mongolia Nepal Central Asia	(Northwest Institute of Plateau Biology, Chinese Academy of Sciences, 1991; DiMa and Mao, 1986; YuTuo, 1982)
*Veronica eriogyne* H.J.P. WinkI.	The whole plant	Bitter, slightly sweet and cool	Internal use: 3∼5 g	Hemostatic and myogenic muscle Stopping bleeding Clearing heat from sores	Growth altitude: 2,200∼4,500 m China (Sichuan, Qinghai, Gansu, Hebei, Shanxi, Shaanxi, Hubei)	(Northwest Institute of Plateau Biology, Chinese Academy of Sciences, 1991; DiMa and Mao, 1986; YuTuo, 1982)
*Corydalis impatiens* (Pall.) Fisch. ex DC.	The whole plant	Bitter and cool	Internal use: decoction, 2–4 g; or into pills or powder. Externally: Appropriate amount, powdered and applied.	Clear heat and reduce swelling Invigorate blood circulation and dissipate blood stasis Treatments for heat-related illnesses Treatment of cold and flu Cure bruises and sores External treatment of boils and poisons	Growth altitude: 1,700 m China (Tibetan Plateau, Sichuan, Gansu, Neimenggu, Shanxi) Russia Mongolia	(Mao, 2012a; Mao, 2016)
*Corydalis crispa* Prain.	The whole plant	Bitter and cool	Internal	Clear heat and removing toxins Relieve pain Cooling the blood	Growth altitude: 3,100∼5,100 m China (Tibetan areas besides Ali and Qiangtang) Western Bhutan	The Tibet Autonomous Region Food and Drug Administration (2012)

### 3.3 Compound recipes and usage

In clinical practice, it is often combined with other Tibetan medicinal botanical drugs for the treatment of inflammatory and hepatobiliary diseases and three-cause diseases. As shown in Table 3, we have compiled 92 preparations containing baxiaga by reviewing the *Four Medical Classics* (YuTuo, 1982), *New Crystal Beads* (Lou, 2004), and other literature works. The main diseases treated by these compound recipes include fever, Chiba disease, Mubu disease, hypertension (Chalong disease), hepatobiliary diseases, and polycythemia (Chapei disease). Bawei Zhuyao San is effective for a variety of internal heat diseases, such as hepatobiliary fever, pulmonary fever, and distemper. Liuwei Muxiang Wan, which combines *Inula ertiliz* Hook. F., *Phyllanthus emblica* L., *Myristica fragrans* Houtt., *C. impatiens*, *Punica granatum* L., and *Piper longum* L., is used to treat gastric ulcers, gastrointestinal pain due to Mubu disorder, acute abdominal pain, belching, bloating, and vomiting (Chen and Gesang, 2017; Danzeng, 2019; GeEr, 1999; Renzhen et al., 2020).

**TABLE 3:** Tibetan medicine prescriptions containing Baxiaga and their functions and indications.

Disease type	Prescription	Medicinal plant	Disease type	Prescription	Medicinal plant
Three-cause diseases	Sanshiwuwei Chenxing San	*Corydalis impatiens* (Pall.) Fisch. ex DC.	Hepatobiliary disease	Jiuwei Niuhuang Wan	Popona
	Zhenlong Xinnao Jiaonang	*Corydalis impatiens* (Pall.) Fisch. ex DC.		Mijue Qingliang San	Baxiaga
	Erhsiwuwei Zhangyacai Wan	*Corydalis impatiens* (Pall.) Fisch. ex DC.		Niuhuang Liniao San	Baxiaga
	Niuhuang Dida Wan	Baxiaga		Sanshiyiwei Songshi Wan	*Corydalis impatiens* (Pall.) Fisch. ex DC.
	Niuhuang Qingpeng Wan	Baxiaga		Shisanwei Niuhuang San	Baxiaga
	Shisanwei Bangga San	*Corydalis impatiens* (Pall.) Fisch. ex DC.		Siwei Yanjing Yaohu	*Justicia adhatoda* L.
	Shisanwei Dida San	Baxiaga		Wuwei ZhaxunTang San	*Corydalis impatiens* (Pall.) Fisch. ex DC.
	Shisanwei Zhangyacai San	*Corydalis impatiens* (Pall.) Fisch. ex DC.		Wuwei Zhangyacai Tang	*Justicia adhatoda* L.
	Dayuejing Wan	*Corydalis impatiens* (Pall.) Fisch. ex DC.	Gastrointestinal disease	Ershijiuwei Nengxiao Wan	Baxiaga
	Ershiliuwei Poxue San	Baxiaga		Mupeng Wan	Baxiaga
	Ershiwuwei Datang San	Baxiaga		Ershiwuwei Mabao Wan	*Corydalis impatiens* (Pall.) Fisch. ex DC.
	Ershiwuwei Hanshuishi San	Baxiaga		Muxiang Daoxiao Wan	Baxiaga
	Ershiyiwei Hanshuishi Wan	Baxiaga		Qiwei Gaoayuanmaogeng Wan	Popona
	Liuwei Muxiang Wan	*Corydalis impatiens* (Pall.) Fisch. ex DC.		Shijiuwei Caoguo San	*Justicia adhatoda* L.
	Shibawei Daxiang Honghua San	Popona		Shiwuwei Muxiang Wan	Baxiaga
	Shiqiwei Hanshuishi Wan	*Corydalis impatiens* (Pall.) Fisch. ex DC.		Shiyiwei Hanshuishi San	Baxiaga
	Shisanwei Muxiang San	Popona		Shiliu Shushi Wan	Baxiaga
	Siwei Shihu Tang	*Corydalis impatiens* (Pall.) Fisch. ex DC.		Songshi Dapeng Wan	Baxiaga
Blood diseases	Zang jiangzhi	*Corydalis impatiens* (Pall.) Fisch. ex DC.	Huangshui disease	ErShisanwei Ercha Wan	Baxiaga
	Dingxiagn Xuebing Wan	Baxiaga		Ershiwuwei Lvxue Wan	Popona
	Ershiwuwei Yuganzi Wan	*Corydalis impatiens* (Pall.) Fisch. ex DC.		Ershiyiwei Ercha Wan	Baxiaga
	Maqian Jiangxue Wan	Baxiaga		Liuwei Kuanjigteng San	Popona
	Qiwei Xuebing Wan	Baxiaga		Shibawei Dangshen San	*Corydalis impatiens* (Pall.) Fisch. ex DC.
	Shibawei Jiangxiang Wan	Baxiaga		Shibawei Ruxiang Wan	*Justicia adhatoda* L.
	Shisanwei Zhixue San	Baxiaga		Shiwei Anxixiang San	*Corydalis impatiens* (Pall.) Fisch. ex DC.
	Shiyiwei Hezi Qingxue San	Baxiaga		Shiwei Ruxiang San	Baxiaga
	Siwei Zhangyacai Tang	*Corydalis impatiens* (Pall.) Fisch. ex DC.		Shiwuwei Rupeng Wan	Baxiaga
​	Wuwei Yuganzitang San	Baxiaga	Nephropathy	Ershiwiwei Xiaoyelian Wan	Baxiaga
	Xuesao Puqing San	*Corydalis impatiens* (Pall.) Fisch. ex DC.		Ershiwuwei Guijiu Wan	*Corydalis impatiens* (Pall.) Fisch. ex DC.Fisch.
	Yazuihua Duwei Tang	*Justicia adhatoda* L.		Honghua Ruyi Wan	Baxiaga
Yutuo Shiwei HongseTang San	Baxiaga	Shiqiwei Huangjing Wan		Baxiaga	​
Lung diseases	Ershiwuwei Feibing San	Baxiaga		Wuwei SazengTang San	Baxiaga
	Erhiwuwei HufeiSan	*Corydalis impatiens* (Pall.) Fisch. ex DC.		Ershibawei Binglang Wan	Popona
	Ershiwuwei Lujiao Wan	*Corydalis impatiens* (Pall.) Fisch. ex DC.		Qiwei Honghua Xiaozhong Wan	*Corydalis impatiens* (Pall.) Fisch. ex DC.
	Liuwei Chuanxixiaohuanju Tang	*Justicia adhatoda* L.	Gynecological diseases	Qiwei Zicaorong Wan	Popona
	Longdanhua Wan	Baxiaga		Shibawei Hezi Liniao Wan	*Corydalis impatiens* (Pall.) Fisch. ex DC.
	Niuhuang Jiangxiang Wan	Baxiaga		Shiliuwei Malinzi Wan	Baxiaga
	Shierjiawei Yishou Wan	Baxiaga		Shisanwei Malin San	*Corydalis impatiens* (Pall.) Fisch. ex DC.
	Shisanwei Zhuhuang San	*Justicia adhatoda* L.		Shisanwei Ximi Wan	*Corydalis impatiens* (Pall.) Fisch. ex DC.
	Shiwuwie Longdanhua Wan	*Corydalis impatiens* (Pall.) Fisch. ex DC.	Other types of diseases	Bawei Zhuyao San	Popona
Hepatobiliary disease	Bawei Xihonghua Qingganre San	*Corydalis impatiens* (Pall.) Fisch. ex DC.		Qiwei Kuanjinteng Tang	*Justicia adhatoda* L.
	Ershiwuwei Jinyaocao Wan	Baxiaga		Liurui San	Popona
	Ershiwuwei Lvronghao Wan	*Corydalis impatiens* (Pall.) Fisch. ex DC.		Mingmu Jiari Wan	​
	Ershiwuwei Songshi Wan	*Justicia adhatoda* L.		Shiwuwei Duocha Wan	Baxiaga
	Ganbing ZongTang San	Baxiaga		Shiwei Muxaing San	Baxiaga
	Jiuwei Niuhuang Kaca Wan	Baxiaga		Jingang Xiaoyan Wan	Popona

### 3.4 Entourage effects in formulations containing Baxiaga

Modern pharmacological research consistently confirms that both Baxiaga as a single botanical drug and formulations containing it are primarily used to treat Tripa disorders in Tibetan medicine theory, which in modern medicine often refer to inflammatory diseases and digestive system disorders represented by hepatobiliary diseases (Quzhen, 2025). However, through combination with other medicinal substances, formulations containing Baxiaga are also employed to treat pulmonary, renal, and gynecological diseases. We propose that this relies on the entourage effects between Baxiaga and other botanical drugs. For instance, the formulation Shiwuwei Longdanhua Wan, which combines Baxiaga with *Gentiana algida* Pall. and botanical drug, is used to treat pulmonary disorders. From a modern pharmacological perspective, its potential synergistic mechanisms can be deconstructed as follows: Baxiaga (inhibiting inflammatory), *Glycyrrhiza uralensis* Fisch. (corticosteroid-like effects), and *Gentiana algida* Pall. (modulating immune function) collectively establish a multi-dimensional anti-inflammatory network targeting cytokines, the hormonal system, and immune cells (Yang et al., 2017; Liu et al., 2017). Furthermore, *Terminalia chebula* Retz. in this formulation is rich in tannins, and studies indicate that certain tannin components can inhibit P-glycoprotein (Do Nascimento et al., 2025). This may delay or enhance the intestinal absorption of key alkaloids from Baxiaga, allowing them to enter the systemic circulation more effectively. More importantly, glycyrrhizic acid derived from *Glycyrrhiza uralensis* Fisch. in this compound acts as a modulator of hepatic cytochrome P450 enzymes (Lin et al., 2022). It may inhibit the rapid hepatic metabolism of active constituents from Baxiaga, reduce their first-pass effect, and thereby increase their systemic availability and duration of action. This dual synergy—both pharmacodynamic and pharmacokinetic—may represent a fundamental scientific principle underlying the efficacy of Tibetan medicinal compounds in treating complex diseases.

### 3.5 Distributions

The majority of these plants are native to China, and their distribution is concentrated on the Qinghai–Tibet Plateau and in the provinces of Sichuan, Yunnan, and Gansu. Beyond China, some of these plants are also found in India, Bhutan, and other regions (Table 2). *J. adhatoda*, which is native to the Indian Peninsula, Mexico, and other tropical areas, is mainly distributed in India, Nepal, Pakistan, Myanmar, Bangladesh, Central America, and other countries or regions. It has also been introduced to Guangdong, Guangxi, Hainan, and Yunnan provinces in China. *V. ciliata* and *V. eriogyne* grow on slopes, meadows, riverbanks, and thickets at altitudes ranging from 2,500 m to 4,500 m. Both species are found in Tibet, Sichuan, Qinghai, and Gansu provinces. *V. ciliata* is also found in Russia, the Himalayas, Mongolia, Nepal, and parts of Central Asia. *C. impatiens* grows at the forest edges, in thickets, or on grassy slopes at altitudes around 1,700 m. It is found in Neimenggu, the Tibetan Plateau, and Sichuan as well as in Mongolia and Siberia. Remarkably, *C. crispa* occurs only in Tibetan areas, including Ali and Qiangtang, and in western Bhutan (https://www.iplant.cn/, Chen and Gesang, 2017; Danzeng, 2019; Geer, 1999; Luo, 2020; Renzhen et al., 2020; Song, 2019; Wangchuk et al., 2012).

## 4 Phytochemistry

A total of 195 compounds have been extracted from baxiaga and include 82 alkaloids (such as quinazoline, isoquinoline and protoberberine alkaloids), 29 flavonoids, 33 terpenoids, 22 phenolic, and 29 additional compound types (Figures 2–9; Table 4).

**FIGURE 2:**
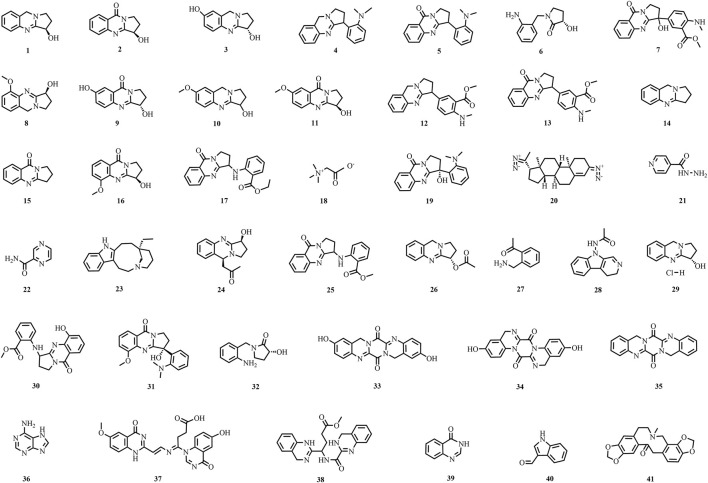
The structures of alkaloids (compound **1**–**41**).

**FIGURE 3:**
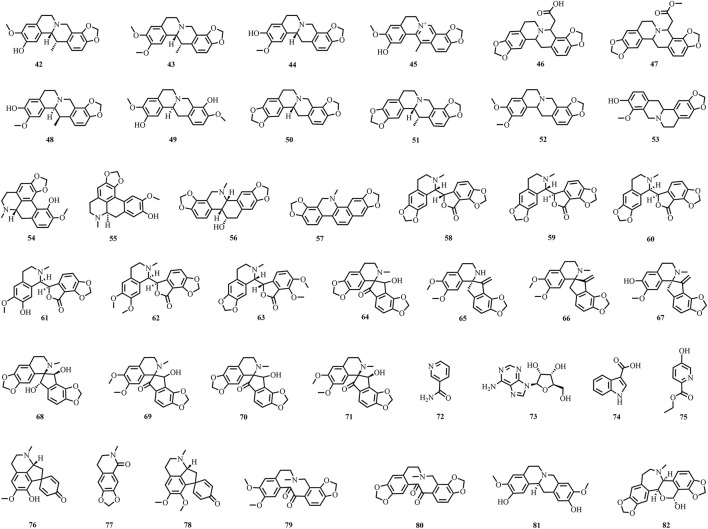
The structures of alkaloids (compound **42**–**82**).

**FIGURE 4:**
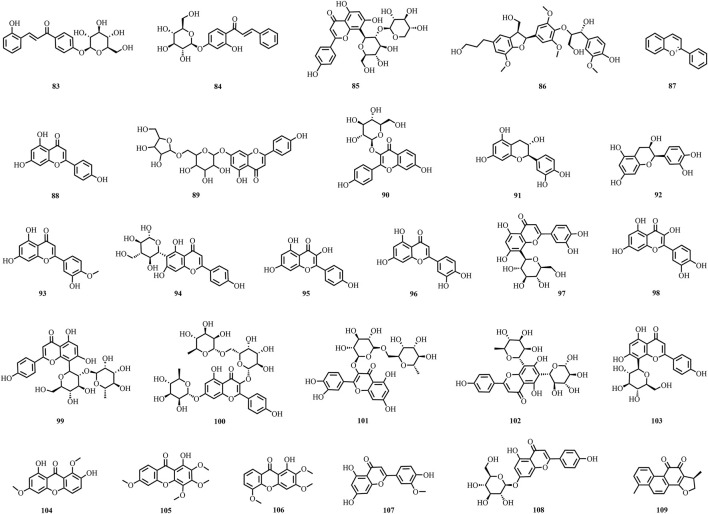
The structures of flavonoids (compound **83**–**109**).

**FIGURE 5:**
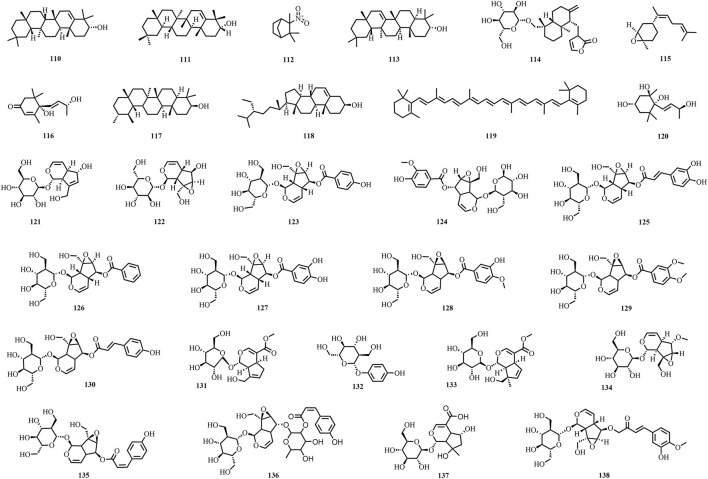
The structures of terpenoids (compound **111**–**138**).

**FIGURE 6:**
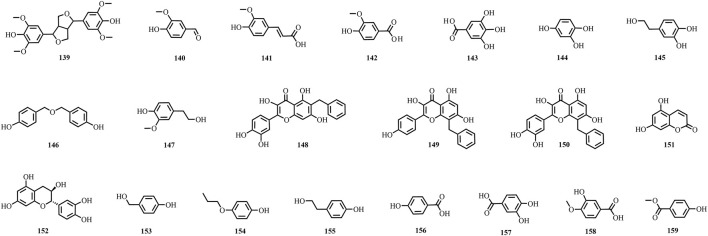
The structures of phenols (compound **139**–**159**).

**FIGURE 7:**
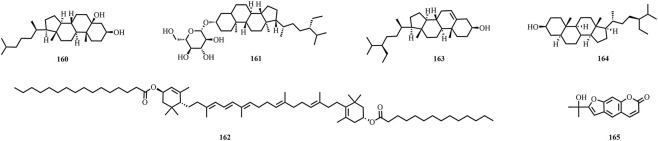
The structures of steroids and phenylpropanoid (compound **160**–**165**).

**FIGURE 8:**
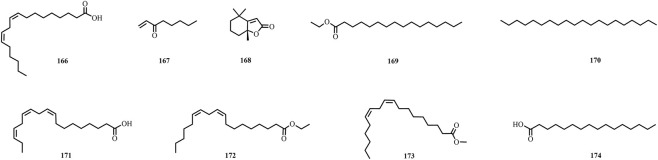
The structures of fatty acids (compound **166**–**174**).

**FIGURE 9:**
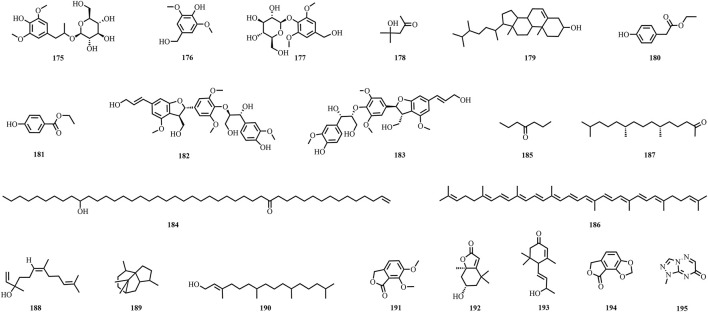
The structures of other types compounds (compound **175**–**195**).

**TABLE 4:** Chemical compounds of Baxiaga.

Chemical class	Compounds	Source plants	References	Structure
Alkaloids	Vasicine	*Justicia adhatoda* L.	(Luo, 2020; Qu et al., 2022 Tehreem et al. (2021))	1
	Vasicinone	*Justicia adhatoda* L.	(Shoaib, 2022 Tehreem et al. (2021))	2
	Vasicinol	*Justicia adhatoda* L.	Tehreem et al. (2021)	3
	Vasicoline	*Justicia adhatoda* L.	(Johne et al., 1971; Shoaib, 2022)	4
	Vasicolinone	*Justicia adhatoda* L.	(Johne et al., 1971; Shoaib, 2022)	5
	Vasicol	*Justicia adhatoda* L.	Luo (2020)	6
	3-Hydroxyanisotine	*Justicia adhatoda* L.	Singh et al. (2011)	7
	5-Methoxyvasicine	*Justicia adhatoda* L.	Singh et al. (2017)	8
	Vasicinolone/7-Hydroxyvascinone	*Justicia adhatoda* L.	(Johne et al., 1971; Shoaib, 2022)	9
	7-Methoxyvasicine	*Justicia adhatoda* L.	(Luo, 2020; Qu et al., 2022; Zhu, 2016)	10
	7-Methoxyvasicinone	*Justicia adhatoda* L.	(Luo, 2020; Qu et al., 2022; Singh and Sharma, 2013)	11
	Adhatodine	*Justicia adhatoda* L.	(Johne et al., 1971; Singh et al., 2011)	12
	Anisotine	*Justicia adhatoda* L.	(Khandelwal et al., 2024; Johne et al., 1971)	13
	Deoxyvasicine/Deoxypeganine	*Justicia adhatoda* L.	Singh et al. (2011)	14
	Deoxyvasicinone	*Justicia adhatoda* L.	Gaur et al. (2025)	15
	Adhavasinone/5-Methoxyvasicinone	*Justicia adhatoda* L.	(Singh et al., 2011; Siva et al., 2022)	16
	Anisessine	*Justicia adhatoda* L.	Mehta et al. (2021)	17
	Btaine	*Justicia adhatoda* L.	Singh et al. (2011)	18
	Desmethoxyaniflorine	*Justicia adhatoda* L.	Singh et al. (2011)	19
	Diazoprogesterone	*Justicia adhatoda* L.	Shoaib (2022)	20
	Isoniazid	*Justicia adhatoda* L.	Rahaman et al. (2018)	21
	Pyrazinamide	*Justicia adhatoda* L.	Rahaman et al. (2018)	22
	Quebrachamine	*Justicia adhatoda* L.	Khan and Bhadauria (2017)	23
	7-Hydroxypeganidine	*Justicia adhatoda* L.	Peron et al. (2024)	24
	Vasnetine	*Justicia adhatoda* L.	Mehta et al. (2021)	25
	Vasicine acetate	*Justicia adhatoda* L.	Duraipandiyan et al. (2015)	26
	2-Acetylbenzylamine	*Justicia adhatoda* L.	Shoaib (2022)	27
	9-acetamido-3,4-dihydropyrido-(3,4-b) indole	*Justicia adhatoda* L.	Mehta et al. (2021)	28
	Vasicine hydrochloride	*Justicia adhatoda* L.	Shoaib (2022)	29
	5-Hydroxy vasentine	*Justicia adhatoda* L.	El-Shanawany et al. (2011)	30
	Aniflorine	*Justicia adhatoda* L.	Siva et al. (2022)	31
​	Vasinol	*Justicia adhatoda* L.	Singh et al. (2011)	32
​	2,10-dihy-droxypyrazino[2,1-b:5,4-b'] diquinazoline-6,14(8H,16H)-dione	*Justicia adhatoda* L.	Zhu (2016)	33
	3,11-dihydroxypyrazino[1,2-a:4,5-a'] diquinazoline-7,15(5H,13H)-dione	*Justicia adhatoda* L.	Zhu (2016)	34
	Pyrazino [2,1 b:5,4-b'] diquinazoline-6,14(8H,16H)-dione	*Justicia adhatoda* L.	Zhu (2016)	35
	Adenine	*Justicia adhatoda* L. *Corydalis impatiens* (Pall.) Fisch. ex DC.	Zhu (2016) Nan et al. (2020a)	36
	(E)-4-(6-hydroxy-4-oxoquinazolin-1(4H) yl)-4-(((E)-2-(6-methoxy-4-oxo-1,4 dihydroquinazolin-2-yl) vinyl) imino) butanoic acid	*Justicia adhatoda* L.	Zhu (2016)	37
	Methyl 4-(1,4-dihydroquinazolin-2-yl)-4-(1,4-dihydroquinazolin-2-carboxami-do) butanoate	*Justicia adhatoda* L.	Zhu (2016)	38
	4(3H)-quinazolinone	*Justicia adhatoda* L.	Qu et al. (2022)	39
	Indole-3-carboxaldehyde	*Justicia adhatoda* L.	Luo (2020)	40
	Protopine	*Corydalis impatiens* (Pall.) Fisch. ex DC. *Corydalis crispa* Prain.	(Wang et al., 2020; Song, 2019) Wangchuk et al. (2012)	41
	Apocavidine	*Corydalis impatiens* (Pall.) Fisch. ex DC.	Niu et al. (2013)	42
	Cavidine	*Corydalis impatiens* (Pall.) Fisch. ex DC.	Niu et al. (2017)	43
	Cheilanthifoline	*Corydalis impatiens* (Pall.) Fisch. ex DC.	(Niu et al., 2013; Song, 2019)	44
	Dehydroapocavidine	*Corydalis impatiens* (Pall.) Fisch. ex DC.	Song (2019)	45
	Impatien B	*Corydalis impatiens* (Pall.) Fisch. ex DC.	Li, J.C. et al. (2013)	46
	Impatien C	*Corydalis impatiens* (Pall.) Fisch. ex DC.	Li et al. (2015)	47
	Isoapocavidine	*Corydalis impatiens* (Pall.) Fisch. ex DC.	(Niu et al., 2013; Song, 2019)	48
	Scoulerine/Discretamine	*Corydalis impatiens* (Pall.) Fisch. ex DC.	Song (2019)	49
	Tetrahydrocoptisine/Stylopine	*Corydalis impatiens* (Pall.) Fisch. ex DC. *Corydalis crispa* Prain.	(Niu et al., 2013; Song, 2019) Wangchuk et al. (2012)	50
	Tetrahydrocorysamine	*Corydalis impatiens* (Pall.) Fisch. ex DC.	Niu et al. (2013)	51
	Tetrahydroepiberberine	*Corydalis impatiens* (Pall.) Fisch. ex DC.	Niu et al. (2013)	52
	Tetrahydrothalifendine	*Corydalis impatiens* (Pall.) Fisch. ex DC.	(Li et al., 2010; Niu et al., 2013)	53
	Bulbocapnine	*Corydalis impatiens* (Pall.) Fisch. ex DC.	Song (2019)	54
​	N-methylactinodaphnine	*Corydalis impatiens* (Pall.) Fisch. ex DC.	Li et al. (2010)	55
	(+) Chelidonine	*Corydalis impatiens* (Pall.) Fisch. ex DC.	Min (2023)	56
	Dihydrosanguinarine	*Corydalis impatiens* (Pall.) Fisch. ex DC.	Song (2019)	57
	Adlumidine	*Corydalis impatiens* (Pall.) Fisch. ex DC.	Song (2019)	58
	Bicuculline	*Corydalis impatiens* (Pall.) Fisch. ex DC. Corydalis crispa Prain.	(Niu et al., 2013; Song, 2019) Wangchuk et al. (2012)	59
	(−) Capnoidine	*Corydalis impatiens* (Pall.) Fisch. ex DC.	(Niu et al., 2013; Song, 2019)	60
	Corlumidine	*Corydalis impatiens* (Pall.) Fisch. ex DC.	Song (2019)	61
	Corlumine	*Corydalis impatiens* (Pall.) Fisch. ex DC.	Song (2019)	62
	Hydrastine	*Corydalis impatiens* (Pall.) Fisch. ex DC.	Min (2023)	63
	Corydaine	*Corydalis impatiens* (Pall.) Fisch. ex DC.	Song (2019)	64
	Norochotensimine	*Corydalis impatiens* (Pall.) Fisch. ex DC.	Li et al. (2010)	65
	Ochotensimine	*Corydalis impatiens* (Pall.) Fisch. ex DC.	Li et al. (2010)	66
	Ochotensine	*Corydalis impatiens* (Pall.) Fisch. ex DC.	Li et al. (2010)	67
	Ochrobirine	*Corydalis impatiens* (Pall.) Fisch. ex DC. *Corydalis crispa* Prain.	Li et al. (2010) Wangchuk et al. (2012)	68
	Raddeanone	*Corydalis impatiens* (Pall.) Fisch. ex DC.	Song (2019)	69
	Sibiricine	*Corydalis impatiens* (Pall.) Fisch. ex DC. *Corydalis crispa* Prain.	Song (2019) Wangchuk et al. (2012)	70
	Yenhusomidine	*Corydalis impatiens* (Pall.) Fisch. ex DC.	Song (2019)	71
	Nicotinamide	*Corydalis impatiens* (Pall.) Fisch. ex DC.	Nan et al. (2020a)	72
	Adenosine	*Corydalis impatiens* (Pall.) Fisch. ex DC.	Nan et al. (2020a)	73
	Indole-3-carboxy acid	*Corydalis impatiens* (Pall.) Fisch. ex DC.	Nan et al. (2020a)	74
	Ethyl-5-hydroxy-2-pyridinecarboxylate	*Corydalis impatiens* (Pall.) Fisch. ex DC.	Nan et al. (2020a)	75
	Glaziovine	*Corydalis impatiens* (Pall.) Fisch. ex DC.	Nan et al. (2020a)	76
​	Oxyhydrastinine	*Corydalis impatiens* (Pall.) Fisch. ex DC.	Song (2019)	77
	Pronuciferine/N-Methylstepharine/Milthanthine	*Corydalis impatiens* (Pall.) Fisch. ex DC.	Song (2019)	78
	13-Oxocryptopine	*Corydalis crispa* Prain.	Wangchuk et al. (2012)	79
	13-Oxoprotopine	*Corydalis crispa* Prain.	Wangchuk et al. (2012)	80
	Coreximine	*Corydalis crispa* Prain.	Wangchuk et al. (2012)	81
	Rheagenine	*Corydalis crispa* Prain.	Wangchuk et al. (2012)	82
Flavonoids	2′-glucosyl-4-hydroxyl-oxychalcone	*Justicia adhatoda* L.	Luo (2020)	83
	2′-hydroxy-4-gluxosyl-oxychalcone	*Justicia adhatoda* L.	Luo (2020)	84
	2″-O-Xylosylvitexin	*Justicia adhatoda* L.	Müller et al. (1993)	85
	Acernikol	*Justicia adhatoda* L.	Luo (2020)	86
	Anthocyanin	*Justicia adhatoda* L.	Sharma et al. (2018)	87
	Apigenin	*Justicia adhatoda* L. *Veronica ciliata* Fisch.	Qu et al. (2022) Lu et al. (2021)	88
	Apigenin-7-O-β-D-apiofruranosyl-(1→6) β-D-glucopyranoside	*Justicia adhatoda* L.	Luo (2020)	89
	Astragalin	*Justicia adhatoda* L.	Singh et al. (2011)	90
	Catechin	*Justicia adhatoda* L.	Basit et al. (2022)	91
	Epi-catechin	*Justicia adhatoda* L.	Basit et al. (2022)	92
	Diosmetin	*Justicia adhatoda* L.	Qu et al. (2022)	93
	Isovitexin	*Justicia adhatoda* L.	Singh et al. (2011)	94
	Kaempferol	*Justicia adhatoda* L. *Corydalis impatiens* (Pall.) Fisch. ex DC.	Qu et al. (2022) Nan et al. (2020b)	95
	Luteolin	*Justicia adhatoda* L. *Veronica ciliata* Fisch.	Qu et al. (2022) Tan et al. (2021)	96
	Orientin	*Justicia adhatoda* L.	Sharma et al. (2018)	97
	Quercetin	*Justicia adhatoda* L. *Corydalis impatiens* (Pall.) Fisch. ex DC.	Qu et al. (2022) Nan et al. (2020b)	98
	Rhamnosylvitexin	*Justicia adhatoda* L.	Luo (2020)	99
	Robinin	*Justicia adhato*da L.	Qu et al. (2022)	100
	Rutin	Justicia adhatoda L.	Basit et al. (2022)	101
	Violanthin	Justicia adhatoda L.	Singh et al. (2011)	102
	Vitexin	Justicia adhatoda L.	Singh et al. (2011)	103
	1,7-dihydroxy-3,8-dimethoxyxanthone	*Corydalis impatiens* (Pall.) Fisch. ex DC.	Chintalwar and Chattopadhyay (2006)	104
	1-hydroxy-2,3,4,6-tetramethoxyxanthone	*Corydalis impatiens* (Pall.) Fisch. ex DC.	Nan et al. (2020b)	105
	1-hydroxy-2,3,5-trimethoxyxanthone	*Corydalis impatiens* (Pall.) Fisch. ex DC.	Nan et al. (2020b)	106
	Chrysoeriol	*Corydalis impatiens* (Pall.) Fisch. ex DC.	Nan et al. (2020b)	107
​	Cynaroside/Luteoloside	*Veronica ciliata* Fisch.	(Gao et al., 2003; Lu et al., 2016)	108
	Dihydrotanshinone I	*Justicia adhatoda* L.	Lu et al. (2021)	109
Terpenoids	3-α-hydroxy-D-friedoolean-5-ene	*Justicia adhatoda* L.	Sultana et al. (2005)	110
	3-α-hydroxy-oleanane-5-ene	*Justicia adhatoda* L.	Sultana et al. (2005)	111
	Bicyclo[2.2.1]heptane, 2,2,3-trimethyl-3-nitro-/2,2,3-Trimethyl-3-nitrobicyclo[2.2.1]heptane	*Justicia adhatoda* L.	Yu et al. (2023)	112
	Epitaraxerol	*Justicia adhatoda* L.	Singh et al. (2011)	113
	Neoandrographolide	*Justicia adhatoda* L.	Kumar et al. (2019)	114
	Trans-Z-α-Bisabolene epoxide	*Justicia adhatoda* L.	Ali et al. (2022)	115
	Vomifoliol/blumenol A	*Justicia adhatoda* L. *Corydalis impatiens* (Pall.) Fisch. ex DC.	Qu et al. (2022) Nan et al. (2020a)	116
	α-Amyrin	*Justicia adhatoda* L.	Singh et al. (2011)	117
	β-Carotene	*Justicia adhatoda* L.	Singh et al. (2011)	118
	γ-Sitosterol	*Justicia adhatoda* L.	Sharma et al. (2018)	119
	Megastigmane	*Corydalis impatiens* (Pall.) Fisch. ex DC.	Nan et al. (2020a)	120
Iridoid glycosides	Aucubin	*Veronica eriogyne* H.J.P. WinkI.	Cui et al. (2014)	121
	Catalpol	*Veronica eriogyne* H.J.P. WinkI.	Cui et al. (2014)	122
	Catalposide	*Veronica ciliata* Fisch. *Veronica eriogyne* H.J.P. WinkI.	Tan et al. (2017) Lu et al. (2016)	123
	Amphicoside	*Veronica ciliata* Fisch. *Veronica eriogyne* H.J.P. WinkI.	Gao et al. (2003) Cui et al. (2015)	124
	Verminoside	*Veronica ciliata* Fisch.	Gao et al. (2003)	125
	Veronicoside	*Veronica ciliata* Fisch.	Lu et al. (2019)	126
	Verproside	*Veronica ciliata* Fisch.	Lu et al. (2016)	127
	6-O-isovanilloylcatalpole	*Veronica ciliata* Fisch.	Gao et al. (2003)	128
	6-O-veratroylcatalposide	*Veronica ciliata* Fisch.	Gao et al. (2003)	129
	6-O-cis-coumarylcatalpol	*Veronica ciliata* Fisch.	Lu et al. (2019)	130
	Geniposide	*Veronica ciliata* Fisch.	Lu et al. (2019)	131
	Specioside	*Veronica ciliata* Fisch.	Lu et al. (2019)	132
	Gardenoside	*Veronica ciliata* Fisch.	Lu et al. (2021)	133
	6-O-methylcatalpol	*Veronica ciliata* Fisch.	Lu et al. (2021)	134
	Arbutin	*Veronica ciliata* Fisch.	Lu et al. (2021)	135
	6-O-(2″-trans hydroxyl trans cinnamic acid rhamnose) catalpol	*Veronica ciliata* Fisch.	Lu et al. (2021)	136
	Acetyl catalpol	*Veronica ciliata* Fisch.	Lu et al. (2021)	137
	Minecoside	*Veronica ciliata* Fisch.	Lu et al. (2021)	138
Phenols	Syringaresinol	*Justicia adhatoda* L.	Luo (2020)	139
	Vanillin	*Justicia adhatoda* L.	Luo (2020)	140
	Ferulic acid	*Justicia adhatoda* L.	Basit et al. (2022)	141
	Vanillic acid	*Justicia adhatoda* L. *Veronica ciliata* Fisch.	Yu et al. (2023) Gao et al. (2003)	142
	Gallic acid	*Justicia adhatoda* L. *Veronica ciliata* Fisch.	Yu et al. (2023) Lu et al. (2021)	143
	1,2,4-Trihydroxybenzene	*Corydalis impatiens* (Pall.) Fisch. ex DC.	Nan et al. (2020b)	144
	3,4-Dihydroxyphenylethanol/Hydroxytyrosol	*Corydalis impatiens* (Pall.) Fisch. ex DC.	Nan et al. (2020b)	145
	4,4′-Dihydroxydibenzyl ether	*Corydalis impatiens* (Pall.) Fisch. ex DC.	Nan et al. (2020b)	146
	4-hydroxy-3-methoxyphenylethanol	*Corydalis impatiens* (Pall.) Fisch. ex DC.	Nan et al. (2020b)	147
	6-C-p-hydroxybenzyl quercetin	*Corydalis impatiens* (Pall.) Fisch. ex DC.	Nan et al. (2020b)	148
	8-C-p-hydoxybenzyl kaempferol	*Corydalis impatiens* (Pall.) Fisch. ex DC.	Nan et al. (2020b)	149
	8-C-p-hydroxybenzylquercetin	*Corydalis impatiens* (Pall.) Fisch. ex DC.	Nan et al. (2020b)	150
	Catechol	*Corydalis impatiens* (Pall.) Fisch. ex DC.	Nan et al. (2020b)	151
	5,7-Dihydroxycoumarin	*Corydalis impatiens* (Pall.) Fisch. ex DC.	Nan et al. (2020b)	152
	p-Hydroxybenzyl alcohol	*Corydalis impatiens* (Pall.) Fisch. ex DC.	Nan et al. (2020b)	153
	p-Hydroxybenzylethyl ether	*Corydalis impatiens* (Pall.) Fisch. ex DC.	Nan et al. (2020b)	154
	p-Hydroxyphenylferulate/Tyrosol	*Corydalis impatiens* (Pall.) Fisch. ex DC.	Nan et al. (2020b)	155
	4-Hydroxybenzoic acid/p-Hydroxybenzoic acid	*Veronica ciliata* Fisch.	(Lu et al., 2016; Tan et al., 2021)	156
	3,4-Dihydroxybenzoic acid/Protocatechuic acid	*Veronica ciliata* Fisch.	(Lu et al., 2016; Tan et al., 2021)	157
	Isovanillic acid	*Veronica ciliata* Fisch.	Gao et al. (2003)	158
	Methyl-p-hydroxybenzoate	*Veronica ciliata* Fisch.	Lu et al. (2019)	159
Steroids	Cholestane-3β,5β-diol	*Justicia adhatoda* L.	Yu et al. (2023)	160
	Daucosterol	*Justicia adhatoda* L. *Corydalis impatiens* (Pall.) Fisch. ex DC.	Singh et al. (2011) Nan et al. (2020a)	161
	Helenien	*Justicia adhatoda* L.	Sharma et al. (2018)	162
	Sitosterol	*Justicia adhatoda* L.	Zhu (2016)	163
	Poriferast-7-en-3beta-ol	*Corydalis impatiens* (Pall.) Fisch. ex DC.	Pan et al. (2017)	164
Phenylpropanoid	1′,2′-Dehydromarmesin	*Justicia adhatoda* L.	Luo (2020)	165
Fatty acid	Linoleic acid	*Justicia adhatoda* L.	Shoaib (2022)	166
	1-Octen-3-one	*Justicia adhatoda* L.	Shoaib (2022)	167
	Dihydroactinidiolide	*Corydalis impatiens* (Pall.) Fisch. ex DC.	Pan et al. (2017)	168
	Ethyl palmitate	*Corydalis impatiens* (Pall.) Fisch. ex DC.	Pan et al. (2017)	169
	Eicosane/Icosane	*Corydalis impatiens* (Pall.) Fisch. ex DC.	Pan et al. (2017)	170
	Linolenic acid	*Corydalis impatiens* (Pall.) Fisch. ex DC.	Pan et al. (2017)	171
	Mandenol	*Corydalis impatiens* (Pall.) Fisch. ex DC.	Pan et al. (2017)	172
	Methyl linoleate	*Corydalis impatiens* (Pall.) Fisch. ex DC.	Pan et al. (2017)	173
	Palmitic acid	*Corydalis impatiens* (Pall.) Fisch. ex DC.	Pan et al. (2017)	174
Others	2- (4-hydroxy-3,5-dimethoxyphenyl) ethyl-β-D-glucopyranoside	*Justicia adhatoda* L.	Sharma et al. (2018)	175
	3,5-dimethoxy-4-hydroxybenzyl alcohol	*Justicia adhatoda* L.	Sharma et al. (2018)	176
	3,5-dimethoxy-4-hydroxybenzyl alcohol 4-O-β-D-glucopyranoside	*Justicia adhatoda* L.	Singh et al. (2011)	177
	4-hydroxy-4-methyl-2-pentanone	*Justicia adhatoda* L.	Zhu (2016)	178
	5-cholestene-3-ol,24-methyl	*Justicia adhatoda* L.	Singh et al. (2011)	179
	Ethyl 2-(4-hydroxyphenyl) acetate	*Justicia adhatoda* L.	Luo (2020)	180
	Ethylparaben	*Justicia adhatoda* L.	Luo (2020)	181
	Jatrointelignan A	*Justicia adhatoda* L.	Luo (2020)	182
	Jatrointelignan B	*Justicia adhatoda* L.	Luo (2020)	183
	37-hydroxyhexatetracont-1-en-15-one	*Justicia adhatoda* L.	Ali et al. (2022)	184
	4-Heptanone	*Justicia adhatoda* L.	El-Sawi et al. (1999)	185
	Lycopene	*Justicia adhatoda* L.	Pan et al. (2017)	186
	Phytone	*Justicia adhatoda* L.	Pan et al. (2017)	187
	*cis*-Nerolidol /1,6,10-Dodecatrien-3-ol,3,7,11-trimethyl-,[S-(Z)]-	*Justicia adhatoda* L.	Krishnamoorthy (2015)	188
	Patchoulane/1H-3a,7-Methanoazulene, octahydro-1,4,9,9-tetramethyl-	*Justicia adhatoda* L.	Sharma et al. (2018)	189
	3,7,11,15-Tetramethyl-2-hexadecen-1-ol	*Justicia adhatoda* L.	Rahaman et al. (2018)	190
	Meconine	*Corydalis impatiens* (Pall.) Fisch. ex DC.	Pan et al. (2017)	191
	Loliolide	*Corydalis impatiens* (Pall.) Fisch. ex DC.	Pan et al. (2017)	192
	9-hydroxy-4,7-megastigmadien-3-one	*Corydalis impatiens* (Pall.) Fisch. ex DC.	Nan et al. (2020a)	193
	Coryhumolide	*Corydalis impatiens* (Pall.) Fisch. ex DC.	Nan et al. (2020a)	194
	1-methyl- [1,2,4] triazolo[4,3-b] [1,2,4] triazin-7-one	*Corydalis impatiens* (Pall.) Fisch. ex DC.	Nan et al. (2020a)	195

### 4.1 Alkaloids

Alkaloids are a class of naturally occurring, nitrogen-containing organic compounds that exhibit basic properties. They typically possess complex ring structures, which often contain nitrogen, and are known for their significant biological activities. Among the five plants studied, alkaloids are the most prevalent substances. A review of previous literature led to the identification of 82 alkaloids (**1**–**82**), including quinazoline, isoquinoline, protoberberine alkaloids, and so on.

Forty alkaloids (**1**–**40**) were isolated from *J. adhatoda*; the main structural types are quinazoline alkaloids, and the main alkaloids are vasicine (**1**), vasicinone (**2**), vasicinol (**3**), vasicoline (**4**), vasicolinone (**5**), vasicol (**6**), 3-hydroxyanisotine (**7**) etc., and 16 other alkaloids (**8**–**16**) (Shoaib, 2022; Khandelwal et al., 2024; Johne et al., 1971; Luo, 2020; Qu et al., 2022; Shoaib, 2022; Singh and Sharma, 2013; Singh et al., 2017; Singh et al., 2011; Gaur et al., 2025; Siva et al., 2022; Tehreem et al., 2021; Zhu, 2016). From stems, leaves and flowers, 11 (**17**–**26**) alkaloids were isolated, among which anisessine (**17**) were firstly isolated (Peron et al., 2024; Duraipandiyan et al., 2015; Khan and Bhadauria, 2017; Mehta et al., 2021; Rahaman et al., 2018; Shoaib, 2022; Singh et al., 2011). Three constituents were isolated from the roots (**27**–**29**), in addition 5-hydroxyvasentine (**30**), aniflorine (**31**), vasinol (**32**), were also isolated from *J. Adhatoda* (El-Shanawany et al., 2011; Mehta et al., 2021; Shoaib, 2022; Singh et al., 2011; Siva et al., 2022). Three new quinazoline alkaloids, namely, 2,10-dihydroxypyrazino[2,1-b:5,4-b’]diquinazoline-6,14(8H,16H)-dione (**33**), 3,11dihydroxypyrazino[1,2-a. 4,5-a’]diquinazoline-7,15(5H,13H)-dione (**34**), and pyrazino[2,1-b:5,4-b’]diquinazoline-6,14(8H,16H)-dione (**35**), were isolated from the stem of *J. adhatoda*; this part also has adenine (**36**), I-4-(6-hydroxy-4-oxoquinazolin-1(4H)-yl)-4-((I-2-(6-methoxy-4-oxo-1,4-dihydroquinazolin-2-yl)vinyl)imino)butanoic acid (**37**), methyl4-(14-dihydroquinazolin-2-yl)-4-(14-dihydroquinazoline-2-carboxami-do)butanoate (**38**), and 4(3H)-quinazolinone (**39**) (Qu et al., 2022; Zhu, 2016). An indole alkaloid, namely, indole-3-carboxaldehyde (**40**), was isolated from *J. adhatoda* for the first time (Luo, 2020).

Alkaloids are characteristic metabolites of plants belonging to the genus *Corydalis*; in particular, 43 alkaloids were isolated from two plants of *Corydalis* (**36**, **41**–**82**). Alkaloids isolated from *Corydalis* mainly include isoquinoline alkaloids; of which, 39 were from *C. impatiens*, including protopioid alkaloids: protopine (**41**), protoberberine (**42**–**53**), aporphine isoquinoline (**54**–**55**), phenphenidine (**56**–**57**), phthalein isoquinoline (**58**–**63**), spirobenzyl isoquinolin (**64**–**71**), and other types of alkaloids (**36**, **72**–**78**) (Song, 2019; Nan et al., 2020a; Wang et al., 2020; Niu et al., 2013; Niu et al., 2017; Li et al., 2013; Li et al., 2015; Li et al., 2010; Min, 2023). Among them, 16 alkaloidal metabolites were first discovered from *C. impatiens* and include dehydroapocavidine (**45**), impatien B (**46**), impatien C (**47**), scoulerine (**49**), bulbocapnine (**54**), (+) chelidonine (**56**), dihydrosanguinarine (**57**), adlumidine (**58**), (−)-capnoidine (**60**), corlumidine (**61**), corlumine (**62**), corydaine (**64**), ochrobirine (**68**), raddeanone (**69**), sibiricine (**70**), and yenhusomidine (**71**) (Li et al., 2013; Li et al., 2015; Min, 2023; Nan et al., 2020a; Niu et al., 2013; Song, 2019). Ethyl-5-hydroxy-2-pyridinecarboxylate (**75**) was isolated as a new compound from *C. impatiens* (Nan et al., 2020a). Protopine (**41**), stylopine (**50**), bicuculline (**59**), ochrobirine (**68**), sibiricine (**70**),13-oxocryptopine (**79**), 13-oxoprotopine (**80**), coreximine (**81**) and rheagenine (**82**) were detached from *C. crispa* (Wangchuk et al., 2012).

### 4.2 Flavonoids

*J. adhatoda* also contains a number of flavonoids, and 23 of which have been reported from *J. adhatoda* (**83**–**103**) (Ali et al., 2022; Basit et al., 2022; El-Sawi et al., 1999; Luo, 2020; Müller et al., 1993; Qu et al., 2022; Singh et al., 2011). In addition to the common apigenin (**88**), diosmetin (**93**), kaempferol (**95**), luteolin (**96**), quercetin (**98**), robinin (**100**), and rutin (**101**) were isolated for the first time (Qu et al., 2022; Basit et al., 2022). Apigenin-7-*O*-*β*-D-apiofruranosyl-(1→6)-*β*-D-glucopyranoside (**89**) was also first isolated from stems and leaves (Luo, 2020). At present, six flavonoids were isolated and identified from *C. impatiens*; these compounds are kaempferol (**95**), quercetin (**98**), 1,7-dihydroxy-3,8-dimethoxyxanthone (**104**), 1-hydroxy-2,3,4,6-tetramethoxyxanthone (**105**), 1-hydroxy-2,3,5-trimethoxyxanthone (**106**), and chrysoeriol (**107**) (Nan et al., 2020b; Chintalwar et al., 2006). Apigenin (**88**), luteolin (**96**), cynaroside (**108**), and dihydrotanshinone I (**109**) were isolated from the ethyl acetate site of *V. ciliata* (Gao et al., 2003; Lu et al., 2021; Lu et al., 2016; Tan et al., 2021).

### 4.3 Terpenoids

Terpenoids are organic compounds, and their derivatives are derived from mevalonate and are composed of isoprene units as the fundamental structural building blocks of their molecular backbone. Our literature review has compiled twenty-nine terpenoid compounds (**110–138**), including iridoid glycosides. Among them, ten terpenoid constituents were identified from *J. adhatoda* (**110**–**119**) (Singh et al., 2011; Rahaman et al., 2018; Khan et al., 2017; Ali et al., 2022; Müller et al., 1993; Sultana et al., 2005; Yu et al., 2023; Kumar et al., 2019). Vomifoliol (**116**) and megastigmane (**120**) were first isolated from *C. impatiens* (Nan et al., 2020a). Iridoid glycosides are the major active constituents of *Brachiaria* spp. Altogether, 18 iridoid glycoside compounds were isolated and identified from *V. ciliata* and *V. eriogyne* and used as baxiaga (**121**–**138**) (Lu et al., 2021; Tan et al., 2017; Lu et al., 2016; Cui et al., 2014; Cui et al., 2015; Lu et al., 2019). Three metabolites, namely, aucubin (**121**), catalpol (**122**), and amphicoside (**124**), were identified from *V. eriogyne* (Cui et al., 2015; Cui et al., 2014).

### 4.4 Phenols

Five phenolic compounds, namely, syringaresinol (**139**), vanillin (**140**), ferulic acid (**141**), vanillic acid (**142**), and gallic acid (**143**), were obtained from *J. adhatoda* (Basit et al., 2022; Luo, 2020; Yu et al., 2023). Some scholars separated and identified 12 phenolic compounds from *C. impatiens* (**144**–**155**); of which, 1,2,4-trihydroxybenzene (**144**), 3,4-dihydroxyphenylethanol (**145**), and 4,4′-dihydroxydibenzyl ether (**146**) were detected for the first time from this plant (Nan et al., 2020b). Seven compounds were detected from *V. ciliata* (**142**, **143**, **156**–**159**) (Lu et al., 2021; Gao et al., 2003; Lu et al., 2016; Cui et al., 2014; Cui et al., 2015; Lu et al., 2019).

### 4.5 Other types

Baxiaga also has 36 other types compounds (**160**–**195**), which include five steroids (**160**–**164**) (Singh et al., 2011; Zhu, 2016; Nan et al., 2020a, Sharma et al., 2018; Kumar et al., 2019; Pan et al., 2017), one phenylpropanoid (1′,2′-dehydromarmesin, **165**) (Luo, 2020), nine compounds from the fatty acid group (**166**–**174**) (Shoaib, 2022; Krishnamoorthy, 2015), and 21 other compounds (**175**–**195**) (Luo, 2020; Shoaib, 2022; Zhu, 2016; Nan et al., 2020a, Sharma et al., 2018; Krishnamoorthy, 2015). Jatrointelignan A **(182)** and jatrointelignan B (**183**) were recovered from the dichloromethane site of *J. Adhatoda* (Luo, 2020).

## 5 Pharmacological activities

**FIGURE 10:**
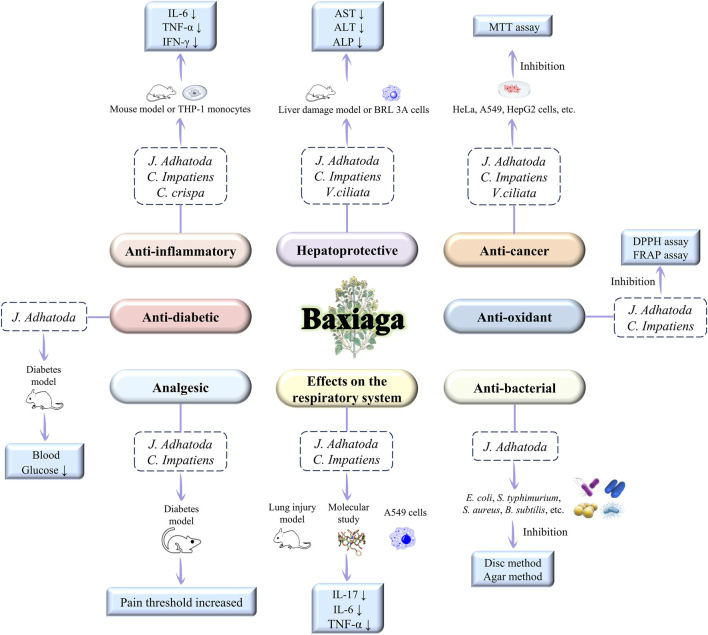
The pharmacological activities of Baxiaga

Baxiaga exhibits several pharmacological effects, which include anti-inflammatory, hepatoprotective, and anticancer (Table 5). Alkaloidal constituents (namely, vasicine **1**, vasicinone **2**, and cavidine **43**), flavonoids, and iridoid glycosides in baxiaga are the major pharmacologically active constituents. Nevertheless, the pharmacological activity of baxiaga plants varies depending on their chemical constituents (Figure 10).

**TABLE 5:** Pharmacological activities of Baxiaga.

Pharmacological activity	Extracts/Compounds	Models/Methods	Dose	Results	Positive control	Experimental duration	Minimum active dose/IC_50_	References
Anti-inflammatory	Aqueous extract of *J. adhatoda*	Mice models of bleomycin induced pulmonary fibrosis and pulmonary sepsis induced by Cecum Ligation and Puncture	130, 260 mg/kg	Inhibits the hallmarks of lung fibrosis and HIF-1α, TGF-β1 and IL-6 levels	Dexamethasone	28 days	130 mg/kg	Gheware et al. (2021b)
	Methanol extract of *J. adhatoda*	Carrageenan and Formalin-induced inflammatory mice models	5, 50,100 mg/kg	Exhibited a significant anti-inflammatory activity against the Carrageenan-induced inflammatory pain	Dexamethasone	8 h	5 mg/kg	Basit et al. (2022)
	Aqueous and alcoholic extracts of *J. adhatoda*	Carrageenan gum-induced paw oedema in rats	500, 1,000 mg/kg	Exhibited marked activity on acute inflammatory process	Dexamethasone	6 h	500 mg/kg	Rajput et al. (2004)
	alkaloid fraction isolated from *J. adhatoda*	LPS-induced inflammation in RAW 264.7 cells	1 ng/mL - 10 μg/mL	Reducing the production levels of TNF-α, IL-6, and ROS	L-NAME	24 h	100 ng/mL	Amala and Sujatha (2021)
	Vasicine and vasicinone extracted from *J. adhatoda*	Carrageenan and CFA-model induced paw oedema	5, 10, 20 mg/kg	Reducing paw edema	Aspirin	6 h 16 days	5 mg/kg	Singh and Sharma (2013)
	Ethyl acetate, n-hexane, and chloroform fractions of *J. adhatoda*	Mice paw edema induced by carrageenan	150, 300 mg/kg	Each fraction demonstrated marked activity at a dosage of 300 mg/kg	Diclofenac	4 h	150 mg/kg	Musa et al. (2022)
	tetrahydrocoptisine isolated from *C. impatiens*	LPS-induced neuroinflammation to mice	18.4, 36.8 mg/kg	inhibiting KMO expression, NO production, and lipid peroxidation; alleviating neuroinflammation	—	16 days	18.4 mg/kg	Khatun et al. (2025)
	Cavidine in *C. impatiens*	Ethanol-induced acute gastric ulcer in mice	1, 5 or 10 mg/kg	Reduces the levels of IL-6 and TNF-α, inhibits the upregulation of COX-2 expression, and suppresses the NF-κB signaling pathway.	Cimetidine	3 h	5 mg/kg	Li et al. (2016)
	Crude extract of *C. crispa*	LPS-activated THP-1 monocytes	50 μg/mL	Inhibiting the experission of TNF-α	Dexamethasone (2013) —	3.5 h	50 μg/mL	(Wangchuk et al., 2012; Wangchuk et al., 2013)
Antioxidant activity and hepatoprotective effect	Methanol extract of *J. adhatoda*	DPPH scavenging assay	10, 20, 30, 40, 50, 60 μg/mL	Significantly reduces DPPH free radicals	Ascorbic acid	30 min	10 μg/mL	Ali et al. (2022)
	Ethyl acetate, chloroform, n-hexane, and aqueous extracts of *J. adhatoda*	DPPH scavenging assay	50, 100 μg/mL	Significantly reduces DPPH free radicals	Ascorbic acid	60 min	50 μg/mL	Musa et al. (2022)
	Extract of J. adhatoda	Aflatoxin B1-induced liver injury model	500 mg/kg	Protects from liver dysfunction induced AFB1	—	10 days	500 mg/kg	Brinda et al. (2013)
	Crude ethanol extract of *J. adhatoda*	Perchloroethylene-induced liver injury in rats	250 mg/kg	Enhance antioxidant enzyme activity, reduce lipid peroxidation, and significantly ameliorate liver injury	Silymarin	10 days	250 mg/kg	Lisina et al. (2011)
	Extract, alkaloid and non-alkaloid parts of *C. impatiens*	CCl4-induced liver injury in mice	1.17, 2.34 g/kg	Alleviates liver injury by inhibiting processes such as lipid peroxidation, oxidative stress, and the release of inflammatory factors.	Polyene Phosphatidylcholine Capsules	7 days	1.17 g/kg	Min (2023)
	n-Butanol extract and iridoid glycosides of *V. ciliata*	ANIT-induced cholestatic liver injury in mice	300, 600, 900 mg/kg 400, 600, 800 mg/kg	Inhibiting oxidative stress and the expression of pro-inflammatory cytokines, while increasing the expression of the proteins NTCP, BSEP, and MRP2	Ursodeoxycholic Acid	14 days	300 mg/kg; 400 mg/kg	Hua et al. (2021)
	Ethyl acetate extract of *V. Ciliata*	BHP induced liver injury in mice	350, 700, 1,400 mg/kg	Reducing oxidative stress and alleviates liver injury	Ursodeoxycholic acid	14 days	700 mg kg	Lu et al. (2021)
	Phenolic and flavonoid derived from ethyl acetate extract of *V. Ciliata*	Ethanol-induced hepatocyte damage	40, 80 μg/mL (flavonoid) 5, 20, 80 μM (phenoli)	Activating AMPK/p62/Nrf2 pathway	—	6 h	40 μg/mL; 5 μM	Lu et al. (2021)
​	Ethyl acetate extracts of *V. ciliata*	Acetaminophen-induced hepatotoxicity in mice	300, 600, 900 mg/kg	Reducing liver function enzyme activity, inhibiting lipid peroxidation, increasing T-AOC and antioxidant enzyme activity, and reversing liver injury	Bifendate pills	14 dasy	600 mg/kg	Lu et al. (2019)
	Ethyl acetate extracts of *V. ciliata*	Acetaminophen in BRL-3A cells	3.75–120 μg/mL	Activating p62- Keap1-Nrf2 pathway	—	14 h	100 μg/mL	Lu et al. (2019)
	Phenols fragment of *V. ciliata*	T-BHP-induced liver injury in BRL 3A cells	15, 30 μg/mL	Inhibition of the disturbed activation of PI3K-Akt signaling pathway	—	8 h	15 μg/mL	Sun et al. (2020)
	Iridoid glycosides frac tion isolated from *V. ciliata*	Acetaminophen-induced hepatotoxicity in mice	150, 300, 450 mg/kg	Noticeable protective effect against acute liver injury	Bifendate pills	14 days	150 mg/kg	Tan et al. (2017)
Anti-asthmatic	Vasicine form *J. adhatoda*	Ovalbumin-sensitized BALB/c mice	25, 50, 100 mg/kg	Modulate the FcεRI/Lyn + Syk/MAPK pathway	Dexamethasone	29 days	25 mg/kg	Qu et al. (2026)
	*J. adhatoda* aqueous extract	Acute allergic asthma in mice sensitised by Ovalbumin injection for 1–3 weeks	13, 65, 130, 195, 260 mg/kg	Reducing IL-4, IL-5, and IL-13 levels, restoring PHD2 expression, and alleviating asthma	Dexamethasone	28 days	13 mg/kg	Gheware et al. (2021b)
	*J. adhatoda* aqueous extract	Dimethyloxaloylglycine induces hypoxia in BEAS-2B cells	10 μg/mL	Inhibiting HIF-1α and improving mitochondrial function	—	32 h	10 μg/mL	Gheware et al. (2021a)
	Cavidine in *C. impatiens*	LPS-induced ALI mouse model	1, 3, 10 mg/kg	Inhibiting of TNF-α and IL-6 production and NF-κB signaling pathway activation	Dexamethasone	6 h	3 mg/kg	Niu et al. (2017)
Antibacterial and antimicrobial	water extracts of *J. adhatoda*	Disc diffusion technique	19.315 g/mL	Inhibiting the growth of *Staphylococcus aureus*	—	—	19.315 g/mL	Fatima et al. (2023)
	Methanolic, ethanolic, and adhatoda-ethyl extracts of *J. adhatoda*	Agar well diffusion method	100, 500, 1,000 μg/mL	Inhibiting the growth of *Aspergillus niger*	Nystatin	7 days	100 ug/mL	Ameer et al. (2023)
	Methanolic and ethanolic extracts of *J. adhatoda*	Agar well diffusion method	100, 500, 1,000 μg/mL	Inhibiting the growth of *Escherichia coli*	Streptomycin sulfate	24 h	100 ug/mL	Ameer et al. (2023)
	Adhatoda-ethyl extracts of *J. adhatoda*	Agar well diffusion method	100, 500, 1,000 μg/mL	Inhibiting the growth of *Staphylococcus aureus*	Streptomycin sulfate	24 h	100 ug/mL	Ameer et al. (2023)
	Phytol isolated and from *J. adhatoda*	Inject ornamental goldfish with a bacterial suspension at a concentration of 1.0 × 10^5^ CFU/mL.	2, 5, 8 mg/kg	Inhibiting the growth of *Bacillus licheniformis*	—	20 days	5 mg/kg	Saha and Bandyopadhyay (2020)
	Silver nanoparticles containing *J. adhatoda* extract	Disc diffusion technique and agar well diffusion method	—	Inhibiting the growth of *Pseudomonas aeruginosa*	Ag^+^	24 h	—	Bose and Chatterjee (2015)
	nanoparticles from *J. adhatoda*	Disc diffusion technique	25, 50, 100 mg/mL	Inhibiting the growth of *Staphylococcus aureus* and *Escherichia coli*	—	24 h	25 mg/mL	Nithya and Sundrarajan (2020)
	*J.adhatoda* extract to assist Ag-Au/ZnO *nanoparticles*	Agar well diffusion method	—	Inhibiting the growth of *Staphylococcus aureus* and *Escherichia coli*	—	24 h	—	Pandiyan et al. (2019)
Anti-cancer	*J.adhatoda* extract to assist Ag-Au/ZnO nanoparticles	HeLa cell	6.25, 12.5, 25, 50 and 100 μg/mL	Inhibited cell growth in a dose-dependent manner	—	48 h	—	Pandiyan et al. (2019)
	Ag-Au/CeO2 nanoparticles prepared from leaf extracts of *J. adhatoda*	HeLa cell	6.25, 12.5, 25, 50, 100 μg/mL	Inhibited cell growth in a dose-dependent manner	—	48 h	—	Nithya and Sundrarajan (2020)
	Ethyl acetate extraction of *J. adhatoda*	HeLa cell	18.75, 37.5, 75, 150, 300 μg/mL	Marked decrease in cell viability at 75 μg/mL and 150 μg/mL	—	24 h	176 μg/mL	Sudevan et al. (2019)
	Mathanolic extract of *J. Adhatoda*	HEK-293, RAW 264.7, SHSY-5Y, MCF-7, and A549 cell	50, 100, 150, 200, 250 μg/mL	Significantly inhibiting the proliferation of MCF-7 cells	-	24 h	161.57 μg/mL	Kumar et al. (2022)
​	70% ethanol crude extracts of *J. Adhatoda*	PA1 cell	6.25, 12.5, 25, 50, 100 in 500 μL of 5% DMEM	Indicated antiproliferative and antimetastatic effects on PA1 cells.	—	24 h	107.339 μg/mL	Nikhitha et al. (2021)
	Ethyl, methanol, and aqueous acetate extract of *J. Adhatoda*	HCC-827 cell	10, 50, 100 μg/mL	Time and dose dependent inhibitory effects	—	48 h	—	Chauhan et al. (2019)
	Vasicine **1** of *J. Adhatoda*	A549 cell	6.25, 12.5, 25,50, 100 μg/mL	Significantly inhibited cell proliferation	—	—	46.5 μg/mL	(Rudrapal et al., 2023)
	Megastigmane **120**	SMMC-7721	2.5, 5.0, 10, 20, 40 μmol/L	Moderate inhibitory effect on cell activity	Cisplatin	24 h	13.8 μmol/L	Nan et al. (2020a)
	8-*C*-*p*-hydroxybenzylquercetin **150**	HepG2 cell	2.5, 5.0, 10, 20, 40 μmol/L	Moderate inhibitory effect on cell activity	Cisplatin	24 h	16.8 μmol/L	Nan et al. (2020b)
	8-C-p-hydroxybenzylkaempferol **149**	HepG2 cell	2.5, 5.0, 10, 20, 40 μmol/L	Moderate inhibitory effect on cell activity	Cisplatin	24 h	19.2 μmol/L	Nan et al. (2020b)
	p-hydroxyphenylferulate **155**	SMMC-7721	2.5, 5.0, 10, 20, 40 μmol/L	Weak inhibitory effect on cell activity	Cisplatin	24 h	24.6 μmol/L	Nan et al. (2020b)
	Cheilanthifoline **44**	HepG2 cell	25, 12.5, 6.25 μg/mL	Inhibited the growth of HepG2 cell	—	24 h	18.92 μg/mL	Song (2019)
	Glaziovine **76**	HepG2 cell	25, 12.5, 6.25 μg/mL	Inhibited the growth of HepG2 cell	—	24 h	2.14 μg/mL	Song (2019)

### 5.1 Anti-inflammatory activity

In Tibetan medical theory, Baxiaga is commonly used to treat heat syndromes and swellings caused by excessive “Tripa” (Zhuoma, C., 2025). Modern research provides a molecular-level explanation. *J. adhatoda* can ameliorate lung fibrosis and airway inflammation, restore the levels of transforming growth factor-β1, IL-6, and HIF-1α in murine models, and reduce the viral load in Vero cells model of SARS-CoV2 infection, indicating the potential of *J. adhatoda* for treatment of COVID-19 (Gheware et al., 2021a). The leaf extract of *J. adhatoda* exhibited significant anti-inflammatory potential in mice induced by carrageenan and formalin (Basit et al., 2022). The water and alcohol extract of *J. adhatoda* exhibited a significant anti-inflammatory action in rat paw edema produced by injecting carrageenin compared with the standard drug diclofenac (Rajput et al., 2004). The arachidonic acid (AA) metabolic pathway plays a critical role in inflammation. Pyrroloquinoline alkaloids present in *J. adhatoda* have been shown to mitigate inflammatory responses by downregulating the expression of inflammatory factors and reactive oxygen species, among others, in LPS-stimulated RAW 264.7 macrophages (Amala and Sujatha, 2021). Some scholars compared the anti-inflammatory activity of most bioactive phytochemicals in chloroform fractions of vasicine **1**, vasicinone **2**, vasicine acetate **26**, 2-acetylbenzylamine **27**, and vasicinolone **9** from *J. adhatoda*; they discovered that vasicine **1** displayed the maximum anti-inflammatory activity in the rat paw edema induced by carrageenan, and vasicinone **2** exhibited the most inhibition at 4 days after complete Fuchs’ adjuvant injection (Singh and Sharma, 2013). In the carrageenan-induced paw edema test, four fractions (ethyl acetate, chloroform, n-hexane, and aqueous) demonstrated anti-inflammatory activity (Musa et al., 2022). Tetrahydrocoptisine **50**, a protoberberine compound from *C. impatiens*, exhibits strong anti-inflammatory activity. Research indicates that it can reduce NO and lipid peroxidation levels, thereby alleviating LPS-induced neuroinflammation in mice (Khatun et al., 2025). Total alkaloids from *C. impatiens* were investigated for their anti-inflammatory effects by establishing xylene-induced auricular swelling in mice and foot-plantar swelling in rats as inflammatory models. Cavidine 43 has a protective effect against gastric ulcers by attenuating the gastric mucosal damage and changes in biochemical indices caused by ethanol administration. The underlying mechanisms may be related to stimulation of PGE2, reduction of oxidative stress, inhibition of NF-κB expression, and subsequent reduction of COX-2 and pro-inflammatory cytokinescc (Li et al., 2016). Crude extracts of six plants were found to inhibit the production of pro-inflammatory cytokines (TNF-α) in LPS-activated THP-1 monocytes, with *C. crispa* showing the best activity. In addition, the n-hexane and dichloromethane extracts of *C. crispa* showed the strongest TNF-α inhibitory activity (Wangchuk et al., 2013; Wangchuk et al., 2011).

### 5.2 Antioxidant activity and hepatoprotective effect

Previous studies have demonstrated that methanol, ethyl acetate, chloroform, n-hexane, and aqueous extracts of *J. adhatoda* can scavenge 2,2-diphenyl-1-picrylhydrazyl (DPPH) radicals, exhibiting significant antioxidant capacity (Ali et al., 2022; Musa et al., 2022). *J. adhatoda* extracts were also responsible for the protective effect in acute oxidative liver injury model evoked by perchloroethylene, aflatoxin B1. The attainment of this effect was mediated through the enhancement of antioxidant enzyme activity, thereby restoring the visibly elevated biochemical levels caused by the injury to the normal range (Brinda et al., 2013; Lisina et al., 2011). The protective effects of *C. impatiens* extracts as well as alkaloidal and non-alkaloidal portions against liver injury were investigated using a CCl_4_-induced acute liver injury model. The alkaloid site represented the main active site against acute liver injury, and the mechanism is associated with the reduction of lipid peroxidation and inflammatory factor release and the alleviation of oxidative stress (Min, 2023). The n-butanol extract and iridoid glycosides from *V. ciliata* exert a dose-dependent protective effect against alpha-naphthylisothiocyanate-induced acute intrahepatic cholestasis in mice, significantly reducing the levels of serum biomarkers associated with cholestasis and liver injury. This effect may be related to the regulation of oxidative stress, inflammatory responses, and bile acid transport (Hua et al., 2021). The iridoid glycoside fraction of *V. ciliata* remarkably decreased the level of the inflammatory factor NF-κB, thereby alleviating the inflammatory response and hepatic injury induced by acetaminophen (APAP) exposure. The ethyl acetate parts of *V. ciliata* and its major metabolites, namely, verproside **127** and catalposide **123**, modulated the p62-Keap1-Nrf2 signaling pathway and protected rat hepatic BRL-3A cells from the effects of APAP. Different fractions of *V. ciliata* medicated the protective response to acute liver injury caused by tert-butyl hydroperoxide by downregulating the expression of the PI3K-Akt and AMPK/p62/Nrf2 signalling pathways (Lu et al., 2021; Lu et al., 2019; Sun et al., 2020; Tan et al., 2017).

In Tibetan medicine, liver and gallbladder diseases are categorized under the category of “Tripa disorders.” The root cause of Tripa disorders lies in the gallbladder, primarily triggered by improper diet—such as excessive consumption of salty or sour foods, unclean dietary habits, indigestion, and emotional factors (Liu et al., 2022). Studies have shown that the hepatoprotective effect of Baxiaga may be achieved through its antioxidant and anti-inflammatory mechanisms, such as inhibiting the NF-κB pathway and activating the AMPK/p62/Nrf2 signaling pathway. This provides modern pharmacological support for the traditional Tibetan medical use of Baxiaga to treat liver disorders.

### 5.3 Effects on the respiratory system

As a bronchodilator, *J. adhatoda* has been utilized to treat asthma for centuries. Vasicine **1** derived from *J. adhatoda* alleviated airway hyperresponsiveness in allergic asthma mice, possibly through modulating the FcεRI/Lyn + Syk/MAPK pathway (Qu et al., 2026). The mechanism by which oral administration of aqueous extract of *J. adhatoda* attenuates acute allergic asthma may be to mitigate the disease in asthmatic animals by inhibiting the level of hypoxia-inducible factor-1a through restoration of the expression of prolyl hydroxylase structural domain-2 (Gheware et al., 2021b). Some researchers used a model of lipopolysaccharide-induced acute lung injury to investigate the lung injury protective effects of the alkaloid cavidine **43** in *C. impatiens*; they found that the protective effects of cavidine **43** might be coordinated by downregulating the levels of inflammatory cytokines by obstructing the activation of NF-κB (Niu et al., 2017).

### 5.4 Antimicrobial and antibacterial activity

The aqueous extract and ethyl acetate extract of *J. adhatoda* exhibited significant inhibitory effects against *Staphylococcus aureus*, while the methanol extract showed significant inhibitory activity against *Bacillus licheniformis* and *Escherichia coli*. In fungal culture experiments, the methanol extract, ethanol extract, and ethyl acetate extract all demonstrated the highest antifungal activity against *Aspergillus niger* at a concentration of 1,000 μg/mL (Fatima et al., 2023; Ameer et al., 2023; Saha and Bandyopadhyay, 2020). The other study reported that silver nanoparticles synthesised from *J. adhatoda* extract displayed relatively effective inhibitory effect against *Pseudomonas aeruginosa*, while Ag-Au/ZnO and Ag-Au/CeO_2_ nanoparticles exerted the most effective inhibitory effect against *E. coli* and *S. aureus* (Bose and Chatterjee, 2015; Nithya and Sundrarajan, 2020; Pandiyan et al., 2019).

### 5.5 Anticancer activity

Previous works reported the outstanding anticancer efficacy of *J. adhatoda*-assisted bimetallic nanoparticles (Ag–Au/ZnO and Ag–Au/CeO_2_) in a human cervical cancer (HeLa) cell line (Nithya and Sundrarajan, 2020; Pandiyan et al., 2019). Another team of researchers used the same cells and found that *J. adhatoda* leaves exerted a protective effect against cancer (Sudevan et al., 2019). The anticancer activity of *J. adhatoda* against diverse cell lines was evaluated using MTT assay; the result showed that the extract effectively works on breast cancer cell line MCF-7 with activation protein of apoptosis, leading to apoptosis and obstruction of the NF-κB pathway (Kumar et al., 2022). Moreover, *J. adhatoda* controlled the growth of ovarian cancer cells (PA1) and a human lung epithelial adenocarcinoma cell line (HCC-827) (Chauhan et al., 2019; Nikhitha et al., 2021). Research has reported that the alkaloid vasicine **1** is derived from *J. adhatoda* differentially suppressed the growth of human lung cancer cell line A549 (Rudrapal et al., 2023).

Several scholars investigated the cytotoxic effects of compounds isolated from *C. impatiens* on human hepatocellular carcinoma cells (HepG2 and SMMC-7721) and MCF-7. Cheilanthifoline **44**, glaziovine **76**, 8-*C*-*p*-hydroxybenzylquercetin **150**, and 8-*C-p-*hydroxybenzylkaempferol **149** showed inhibitory effects on the HepG2 model but had no effect on MCF-7; meanwhile, megastigmane **120** and *p*-hydroxyphenylferulate **155** was inhibitory against the hepatic cell line SMMC-7721 (Nan et al., 2020a; Nan et al., 2020b; Song, 2019).

## 6 Toxicology

Toxicity studies on *J. adhatoda* and *C. impatiens* were reported in the literature. The acute toxicity of *J. adhatoda* extract and fresh juice administered to rats at a dose of 2 g/kg was observed with regard to behavioral performance after 24 h; any abnormal behavior in mice after receiving 150–180 mg/kg *J. adhatoda* extract during 72 h was determined. None of the animals exhibited abnormal behavior or died. Chronic toxicity studies were carried out for 14 days by using different doses of *J. adhatoda* extract; mortality or delayed effects were not detected in the animals. Collectively, these findings imply that the administration of different doses of *J. adhatoda* does not produce toxic effects (Musa et al., 2022; Patil, 2010; Sarker et al., 2009). In addition, mice were orally administered with 200, 400, and 600 mg/kg vasicinone **2** for 7 days, and death or toxicity was not observed (Sarkar et al., 2014). Experimental studies were conducted on the acute and subchronic oral toxicity of the total alkaloids of *C. impatiens* on mice. The LD_50_ was 42.9 mg/kg. The subchronic oral toxicity study was carried out in mice by administering 5.0, 10.0, and 20.0 mg/kg total alkaloids of *C. impatiens* for 28 days. After testing the biochemical parameters of each organ, organ indices were calculated and histomorphological changes were monitored. The data from the study revealed that kidney and liver involvement was relatively extensive in mice and was prominent in females than in males (Li et al., 2019). The toxicity of the ethanolic extract of *V. ciliata* was investigated by administering 5 g/kg, which is equivalent to 48.1 g/kg of the raw drug, for 14 consecutive days; the behavior, body weight, water intake, and dietary intake of mice in each group were monitored. Mice in each group had abnormal behavior and sustained increase in body weight, diet, and water intake but had no abnormality changes in major organ indices, serum biochemical indices, and histopathological examination. The mice did not experience any noticeable acute toxicity reactions (Tan et al., 2018).

## 7 Quality control

Pharmaceutical quality is concerned with the safety and efficacy of clinical use of medicines; thus far, limited research has been conducted on the quality control of five plants in baxiaga. As Tibetan medicinal habitual botanical drugs, the five original plants of baxiaga are not registered in the Chinese Pharmacopoeia but are regulated in the standard of local medicinal materials in Tibet, Qinghai, Sichuan, and other provinces. In the existing quality standards for *J. adhatoda*, *V. ciliata*, *V. eriogyne*, *C. impatiens*, and *C. crispa*, studies were concentrated on qualitative identification to describe the traits and characteristics, microscopic identification, and thin-layer identification of the main metabolites of the botanical drugs; however, the contents of the main metabolites were not determined (The Tibet Autonomous Region Food and Drug Administration, 2012; QingHai Medical Products Administration, 2019; SiChuan Medical Products Administration, 2014, 2020). Luo determined the content of vasicine **1**, which is the active ingredient of *J. adhatoda*, and recommended the quality standard based on the results. The content of vasicine **1** in the herb should not be less than 0.46%, which was calculated as a dried product and determined through high-performance liquid chromatography (HPLC) (Luo, 2020). Protopine **41** was used as a quality control index and a and incorporated in a method to determine *C. impatiens* content. The content of protopine **41** in *C. impatiens* should not be less than 0.058% (Li, 2014). Other scholars also determined the total alkaloid content of *C. impatiens* with reference to berberine hydrochloride and reported that the total alkaloid content ranged from 0.87% to 1.02%, with 20% downward adjustment from the mean value; the finding suggested that the total alkaloid content should not be less than 0.7% as a quality standard for *C. impatiens* (Puba et al., 2015). The HPLC-DAD fingerprint study identified 14 peaks in the extract of *C. impatiens*; the similarity of the 10 batches of samples used for analysis was greater than 0.9, and peak No. 10 was identified as protopine **41** and peak No. 13 was bicuculline **59**. Accordingly, a new recommendation was made for the quality standard for *C. impatiens*, that is, the contents should not be less than 0.47% for bicuculline **59**% and 2.29% for protopine **41** in dry form (Chen et al., 2019; Min, 2023).

Scholars have developed methods for the determination of the contents of aucubin **121**, picroside II **131**, and catalpol **122** in *V. eriogyne*. The iridoid glycosides had linear relationships within a certain range. The average contents of aucubin **121** and catalpol **122** were 4.95 and 4.16 mg/g, respectively, and the spiked recoveries (*n* = 6) were 103.8% (RSD = 0.7%) and 98.9% (RSD = 2.1). The results provide a basis for exploring the development of improved quality standards for *V. eriogyne* botanical drugs (Cui et al., 2015; Cui et al., 2014). The levels of four major iridoid glycosides, namely, catalpol **122**, catalposide **123**, picroside II **131**, and 6-*O*-veratroylcatalposide **129**, were simultaneously determined in *V. ciliata* by ultra-high-performance liquid chromatography (UPLC); the results can be used for the quality control of these metabolites (Luo et al., 2019). HPLC profiling methods were established for 10 batches of *V. ciliata* from different origins, and the main chemical metabolites were quantified. All the batches had 16 common peaks; two common peaks were identified to be picroside II **131** and luteolin **96**, and the content of the former ranged from 0.35% to 2.60%. Hence, the method is accurate, reliable, and exclusive and can be used as a reference for the quality evaluation of *V. ciliata* (Wang et al., 2022).

## 8 Discussion

This study systematically reviews the multi-origin plant system of the Tibetan medicine Baxiaga and integrates research on its chemical constituents and pharmacological activities. It confirms that its primary botanical origins involve species such as *J*. *adhatoda*, *V*. *ciliata*, and the *C. impatiens* and systematically summarizes 195 reported chemical metabolites, constructing a chemical profile centered on alkaloids, flavonoids, and iridoids. The research reveals that these core component groups not only serve as the material basis for its “bitter taste and cool nature” as recorded in the Tara Materia Medica (Mao, 2016b), but also function as the key active units responsible for its essential pharmacological activities, including anti-inflammatory, antioxidant, and hepatoprotective effects. This provides a molecular-level explanation for the traditional applications of Baxiaga in clearing heat, detoxifying, and promoting bile flow in Tibetan medical theory.

### 8.1 Differential therapeutic potential of Bxiaga

We analyzed the differences in major pharmacologically active metabolites of Baxiaga derived from various botanical sources. The study revealed that *J. adhatoda* is likely the most robust choice for broad anti-inflammatory and hepatoprotective applications, owing to its comprehensive chemical profile. For more targeted hepatoprotective effects, *V. ciliata*, which is rich in iridoid glycosides, may offer distinct advantages and potential. Meanwhile, *C. impatiens* could possess unique strengths in anti-inflammatory and antioxidant activities due to its specific combination of alkaloids and phenolic compounds. However, the current lack of studies that directly compare the activities of extracts from different Baxiaga sources using the same models and evaluation metrics limits the analysis of activity differences to correlative inferences at this stage.

### 8.2 Limitations

Although this review systematically integrates existing knowledge on Baxiaga, the therapeutic prospects of this medicinal plant still face significant limitations. Firstly, as a multi-origin medicinal plant, baxiaga botanical drugs have complex compositions. The list of active compounds and the interactions of each medicinal material are incompletely documented, causing difficulties in ascertaining the pharmacological activity of the plant. Insufficient knowledge of the specific chemical compositions has also caused difficulties in the research and development of Tibetan medicine. Moreover, modern pharmacological research on it, especially regarding the exploration of molecular mechanisms targeting specific active metabolites, is still in its early stages. Currently, the vast majority of studies concentrate on observing the overall effects at the extract level, lacking in-depth investigation that connects specific chemical constituents with precise molecular targets and signaling pathways. Furthermore, the varieties of Baxiaga used in different regions of Tibet vary in origin, making quality control of the medicinal materials extremely challenging. Additionally, due to the absence of standardized methods, the efficacy evaluation of Baxiaga is often subjective and yields incomparable results.

### 8.3 Future perspective

To address the aforementioned issues, future research should prioritize the following directions.

Optimization of modern analytical techniques to interpret the constituents of baxiaga is an urgent problem to be solved. NMR, FT-IR, and LC-MS have been applied for the structural characterization of alkaloids, flavonoids, iridoid glycosides, and steroidal constituents of baxiaga (Luo, 2020; Nan et al., 2020b; Song, 2019; Tehreem et al., 2021). Studies on the active ingredients of baxiaga have only concentrated on its known quinoline/isoquinoline alkaloids or iridoid glycosides metabolites (Deng et al., 2019; Liu et al., 2015). Future studies should investigate whether flavonoids, steroids, and phenolics in the botanical drugs have certain pharmacological effects and whether the compounds interact with one another. Various mechanisms are involved in the pharmacological action of baxiaga. Different botanical drugs and their active ingredients may exert therapeutic effects through different mechanisms, such as anti-inflammatory, hepatoprotective, and anticancer, leading to complex pharmacological effects.

Bioinformatics tools, such as molecular docking and network pharmacology, should be combined to sufficiently comprehend the biological functions and interactions of baxiaga and select potential active ingredients and targets. Molecular docking, UV-vis spectroscopy, fluorescence spectroscopy, and differential scanning calorimetry have been exploited to reveal the molecular mechanism of the interaction between vasicine **1** and DNA at the physicochemical level (Murali et al., 2017). Some studies reported the combinations of chemical genomics survey, reverse pharmacophore target matching, and comparative molecular docking analysis to predict the potential antimicrobial binding capacity of vasicine **1**, vasicinone **2**, and deoxyvasicine **14** to sixteen antimicrobial targets of *Mycobacterium tuberculosis* (Siva et al., 2022). Furthermore, molecular docking has allowed the prediction of the binding capacity of the alkaloidal active ingredients in *J. adhatoda* to ACE, SARS-CoV-2 protease, breast cancer susceptibility gene 1, cornulin, E3 ubiquitin-protein ligase WWP1, 5-lipoxygenase, and so on (Afzal, 2013; Ghanta et al., 2022; Jayaraman et al., 2020; Selvaraj et al., 2021a; Selvaraj et al., 2021b; Siva et al., 2022; Tehreem et al., 2021). But, these predictions require additional validation in combination with cellular and animal models to reveal the integrated mechanism of drug action through the network regulation of multi-metabolites, multi-targets, and multi-pathways.

Guidelines and methods should be developed to harmonize efficacy assessment, essential active ingredients should be used as biomarkers for quantitative analysis and efficacy assessment, and comparability should be ensured between different studies. The determination of vasicine **1** content in *J. adhatoda* was taken as an example. The content of vasicine **1** in the herb should not be less than 0.46% based on the dried product, as determined by HPLC. The method was further optimized for quantitative analysis using HPLC-DAD, which showed good linearity in the range of 5.125–205 μg/mL with an average recovery of 102.3% and RSD of 4.3% (Luo, 2020; Velichkova et al., 2022). Quality control standards for baxiaga, such as in herb harvesting and processing specifications and fingerprinting, should be improved to ensure consistency. The extraction, concentration, and formulation processes should be optimized to maintain the stability and consistency of the preparations. According to *SiChuanSheng ZangYaoCai BiaoZhun (2020*) and *Tibet Autonomous Region Local Medicinal Materials (Tablets) Quality Standards (2023)*, the original species of baxiaga is *J. adhatoda*, whereas the *QinghaiSheng ZangYaoCai BiaoZhun (2019*) records it as *C. impatiens*. Existing standards have recorded the medicinal parts of botanical drugs, standardized the harvesting and processing of botanical drugs, and formulated the identification standards of botanical drugs; however, content determination is missing (QingHai Medical Products Administration., 2019; SiChuan Medical Products Administration., 2020). The ineffective bioavailability of vasicine **1**, which is caused by impaired solubility, diminished permeability, and malabsorption in the gut, seriously restricted its therapeutic application. Therefore, scholars applied phytophospholipid complexes for developing a nanomedicine delivery system to improve the oral bioavailability of vasicine **1** (Nandhini and Ilango, 2021). Another study developed a new lyophilized formulation that improves the oral bioavailability and stability of vasicine **1** and reduces its conversion into vasicinone **2** (Vyas et al., 2011). An abundance of alkaloids in baxiaga has made an important contribution to the development of Tibetan medicine; among which, quinoline alkaloids, such as vasicine **1** and vasicinone **2**, have been widely used in clinical treatments and are in high demand in the market. Techniques involving biosynthesis, *in vitro* tissue culture, and alteration of environmental UV-B radiation have been used to enhance the conversion and content of quinoline alkaloids (Afanasyev et al., 2020; Pandey et al., 2021; Panigrahi et al., 2017; Richers et al., 2013; Singh et al., 2017; Singh et al., 2022). The *J. adhatoda* extract, which is known as a prospective acetylcholinesterase inhibitor, exhibited remarkable bronchodilation and neuroprotection activities. Novel 3-OH pyrrolidine derivatives have been designed and synthesized by semi-synthetic approaches with vasicine **1** as a precursor. Numerous multi-targeted and multifunctional anti-Alzheimer’s disease compounds with various structural aminoglycosides have been synthesized using deoxyvasicinone **15** (Bhanukiran, 2023; Chen et al., 2021; Ma and Du, 2017). Modern production equipment and technology will be introduced to enhance quality control. Adaptive trial designs will be implemented to improve efficiency and reduce time and costs. Digital health technologies and wearable devices will be used for remote monitoring and data collection to increase patient adherence to baxiaga.

These proposed directions are designed to directly tackle the aforementioned limitations—clarifying material basis, elucidating mechanisms, ensuring quality, and validating efficacy—thereby transforming Baxiaga from a traditionally used complex into a reliably applied modern therapeutic agent.
